# Revealing Interface Polarization Effects on the Electrical Double Layer with Efficient Open Boundary Simulations under Potential Control

**DOI:** 10.1021/acs.jpclett.3c03615

**Published:** 2024-04-29

**Authors:** Margherita Buraschi, Andrew P. Horsfield, Clotilde S. Cucinotta

**Affiliations:** † Department of Chemistry, Imperial College London, White City Campus, London W12 0BZ, U.K.; ‡ Department of Materials, Imperial College London, South Kensington Campus, London SW7 2AZ, U.K.; ¶ Thomas Young Centre, London, U.K.

## Abstract

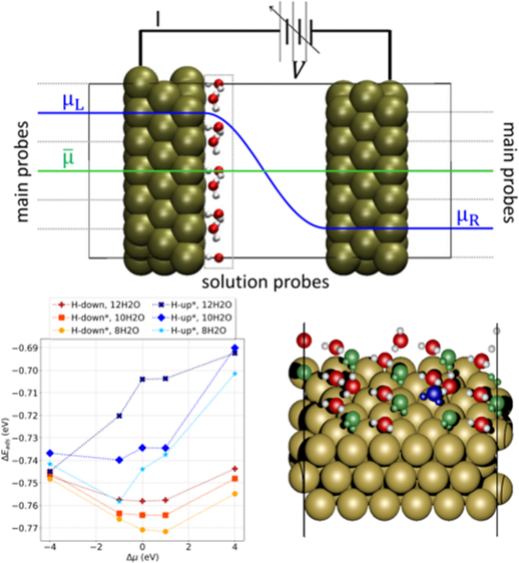

A major challenge in modeling interfacial processes in electrochemical (EC) devices is performing simulations at constant potential. This requires an open-boundary description of the electrons, so that they can enter and leave the computational cell. To enable realistic modeling of EC processes under potential control we have interfaced density functional theory with the hairy probe method in the weak coupling limit (Zauchner et al. Phys. Rev. B2018, 97, 045116). Our implementation was systematically tested using simple parallel-plate capacitor models with pristine surfaces and a single layer of adsorbed water molecules. Remarkably, our code’s efficiency is comparable with a standard DFT calculation. We reveal that local field effects at the electrical double layer induced by the change of applied potential can significantly affect the energies of chemical steps in heterogeneous electrocatalysis. Our results demonstrate the importance of an explicit modeling of the applied potential in a simulation and provide an efficient tool to control this critical parameter.

The functionality of electrochemical (EC) devices is primarily determined by the electrified interface (EI) where the formation of an electrical double layer (EDL) occurs. To achieve a rational design of EC devices, an atomistic understanding of the EDL is highly desirable^1^ as its equilibrium structure and response to applied potential directly affect the thermodynamics and kinetic barriers for interfacial mass and charge transfer (CT).^2^ First-principles computational studies are well suited for this task as they achieve electronic level resolution.^3,4^

The chemical reactivity and structural properties of the EDL are tuned by the electrode potential. However, including the potential in computer simulations remains a challenge.^3,4^ EC systems are often studied using density functional theory (DFT) in the canonical ensemble (constant temperature, volume and number of particles). An elegant approach to incorporate the electrode potential is Nørskov’s computational hydrogen electrode (CHE), which has proven successful in replicating the experimentally well-known volcano plot for hydrogen evolution reaction (HER).^5,6^ However, the potential is implicitly included as a thermodynamic correction only to the electron–proton transfer steps of the reaction. CHE fails to capture the potential-induced charging of the interface under operating conditions as it considers steps such as H_2_O, O_2_ or CO_2_ adsorption as independent of the applied potential. This has recently been proven to be incorrect.^7^

A more realistic way to explicitly introduce the effect of a potential in canonical simulations is to control the charge on the electrode surface and evaluate *a posteriori* the relation between potential and charge.^4^ This is achieved by adding a judicious distribution of counter-charges that enforce cell neutrality.^3,8−19^ Despite the undeniable insight these approaches have provided about the EIs, fixed-charge simulations remain conceptually different from fixed-potential simulations. All of the above approaches need either large samples or repeated simulations,^4^ fictitious distributions of charge, lack of flexibility in varying the electrode potential, and an inability to determine directly the potential dependence of transition states’ charges and activation barriers. Ultimately, the electrode potential is not guaranteed to remain constant during the simulation as charge transfer at the interface causes fluctuations in the electrode charge and, consequently, in its potential.

Grand-Canonical DFT (GC-DFT)^20−23^ allows the number of electrons (*N*_*e*_) in the cell to vary while keeping the chemical potential of the electrons (μ_*e*_) fixed. The grand potential of the system, rather than the free energy, is minimized as a function of the electron density.^24^ This formulation is suitable for describing metallic slabs that form part of an electric circuit. However, GC-DFT simulations are generally harder to converge than canonical ones^21,24,25^ and often use implicit models to describe the electrolyte.^22,26−32^

As the setup of electrochemical cells reflects the computational framework used in molecular electronics, Non-Equilibrium Green’s Function (NEGF) combined with DFT is emerging as a method to calculate the effects of bias on the electronic properties and atomic forces at the EI.^1,33^ While this approach enables a fine-tuned control of the potential in a simulation, the high computational cost of the NEGF+DFT calculations in current implementations makes simulating large systems difficult and expensive.

In this paper, we propose a novel and efficient methodology to introduce electronic open-boundaries into DFT calculations. The hairy probes (HP) formalism^34,35^ is an open-boundaries formalism suitable for multiterminal EC problems. Here we interface HP with the traditional Kohn–Sham DFT formalism, as implemented in CP2K.^36^ The aim is to develop a computational tool, hairy-probes DFT (HP-DFT), which enables electronic structure and force calculations under potential control. HP-DFT proved to be lightweight and highly efficient, showcasing computational costs comparable to that of standard DFT calculations.

The foundation of the HP formalism is the concept of *probe*. Each probe is a virtual, atomically thin lead coupled at one end to an electron reservoir, with a known electrochemical potential (μ_*p*_) and temperature (*T*_*p*_), and at the other end to an orbital of the system. How strongly the probes are coupled to the system is defined by the coupling strength parameter Γ_*p*_. Each probe can act as both a source and sink of electrons at a given μ_*p*_. The probes are assumed to be in equilibrium with their respective reservoir so that the electrochemical potential and temperature of the electrodes correspond to those of the reservoirs they are coupled to.^35^ The HP formalism implemented here allows for any number of probes, making it suitable for multiterminal EC problems.

In the limiting case of probes weakly coupled to the system, Γ_*p*_ → 0, thus it does not appear in the central formulas. It was shown that this weak coupling limit is suitable for describing EC systems^34^ as the charge is carried from one electrode to the other by ions in the electrolyte, whose diffusion rate sets the electron conduction rate between electrodes. Since ion diffusion is much slower than ballistic transport within the probes, it is reasonable to assume that the weakly coupled probes do not restrict current flow.

The probes responsible for maintaining the electrochemical potential difference across the system (*main probes*) are typically coupled only to the atoms in the contact regions. This might result in the situation where atoms located far from the contacts remain uncoupled to the reservoirs, and thus not populated with electrons. Thus, we also implemented a secondary type of probe coupled even more weakly to the remaining atoms (*solution probes*): they have no obvious physical meaning and they simply prevent the nonphysical situation where molecules in solution have no electrons. Thus, the *main probes* coupled to the contact regions are used to induce an EC potential difference across the system while the *solution probes* coupled to the electrolyte’s molecules are needed to ensure the correct population distribution in the solution.

The HP formalism is introduced into the DFT through the general representation of the electron density:
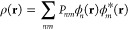
1with
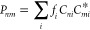
2where ϕ_*n*_(**r**) are the basis functions used to represent the molecular states and *P*_*nm*_ is the density matrix, described by Equation 2, where *C*_*ni*_ are the Molecular Orbital (MO) coefficients and *f*_*i*_ are the occupation numbers of the molecular states. DFT codes often employ the Fermi–Dirac distribution, to define *f*_*i*_. In the weak coupling HP formalism, the occupation numbers are instead given by
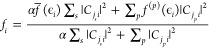
3with
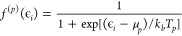
4
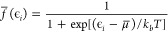
5

In Equation 3, ϵ_*i*_ is the MO energy for orbital *i*,  is the coefficient of MO *i* for the atomic orbital *j*_*p*_ coupled to probe *p*, and *f*^(*p*)^ is a Fermi–Dirac distribution defined by the electrochemical potential μ_*p*_ and electronic temperature *T*_*p*_ of the electrons in the reservoir for probe *p* (Equation 4);  are the MO coefficients of the atoms coupled with the solution probes and  is a Fermi–Dirac distribution defined by μ̅, the average electron electrochemical potential of the system (Equation 5). The level μ̅ is calculated as the average among the local Fermi levels induced by the probes’ EC potential and is the level which ensures charge neutrality in the system. The way we define μ_*p*_ is . Finally, α is a small parameter that allows smooth switching of the electron population between those from the main probes and that for the solution probes. For a full derivation of these formulas, we direct the reader to references^34^ and.^35^

To implement the HP-DFT formalism we developed and included in the QUICKSTEP^37^ module of CP2K^36^ a module to calculate *f*_*i*_ using Equation 3.

We evaluated our HP-DFT implementation using parallel-plate capacitor models, consisting of two Pt(111) slabs separated by 10 *Å*. We tested models with varying surface areas and slab thicknesses, resulting in three supercells, Pt(111)(a × a × b), where a indicates the number of replicas of the orthorhombic unit cell for Pt(111) surface, and b the number of layers in the slab. The three configurations considered are (2 × 2 × 3), (2 × 2 × 5), and (6 × 6 × 3). To model a simple interface, we adsorbed an ice-like water bilayer on the left surface of the Pt(111)(6 × 6 × 3) supercell while the right slab served as counter electrode. Both the H-down and H-up configurations^38,39^ were considered. Our models consist of 24 water molecules: 12 form the first layer of chemisorbed molecules, while the remaining 12 form the second layer. This system is defined as “full coverage”, as 12 is the maximum number of molecules which can be chemisorbed on the 6 × 6 surface for an ice-like H-down and H-up bilayer structure. Additionally, we created “low water coverage structures” by removing 2 and 4 water molecules from the first water layer in both H-up and H-down models. These structures are denoted as 12H2O, 10H2O and 8H2O. For these models, the separation between the two plates was increased to 20 *Å* to avoid interaction with the right surface (these distances are measured from the center of mass of the atoms of the internal surfaces; to calculate the capacitance, *d* was corrected by the van der Waals radius of Pt).

The QUICKSTEP module employs a mixed Gaussian and plane waves (GPW) approach^37,40^ to perform electronic structure calculations. In all our calculations the Perdew–Burke–Ernzerhof (PBE) exchange-correlation functional^41^ was used in combination with the Grimme’s D3 correction for dispersion interactions.^42^ Goedeker-Teter-Hutter (GTH) pseudopotentials^43^ were used to describe the core electrons. TZVP-MOLOPT basis sets^44^ were used to expand the Kohn–Sham orbitals. For the Pt(111)(2 × 2 × 3) and Pt(111) (2 × 2 × 5) systems an 8 × 8 × 1Monkhorst–Pack grid of k-points^45^ was employed to sample the Brillouin zone. For the Pt(111)(6 × 6 × 3) system calculations were performed at the Γ-point. A conventional cubic cell with a lattice parameter of 3.97 *Å* was employed to build the slabs. The introduction of an EC potential difference induces a dipole within the system. For this reason, periodic boundary conditions were applied only in the directions parallel to the surfaces of the plates (x and y), while in the direction perpendicular to them (z) an implicit Poisson solver was used.^46^ Threshold parameters for the SCF and force convergence were set at 1.0 × 10^–7^ E_h_ and 1.0 × 10^–3^ E_h_/a_0_ respectively. The overall error associated with the total energy calculated with these parameters is ±7.35 E_h_/cell.

For the HP-DFT calculations, two sets of main probes were coupled with the outermost layers of the capacitors, as depicted in Figure 1. The applied electrochemical potentials were chosen to satisfy Δμ_*L*_ = −Δμ_*R*_ to give  and . The resulting electrochemical potential difference across the system is Δμ = |μ_*L*_ – μ_*R*_|. In systems with water, solution probes were coupled with each atom of the molecules, as depicted in Figure 1. Because of the way the HP-DFT was implemented, the cell is charge neutral. This ensures that the energy is well-defined. Finally, the probes impose well-defined *electron* electrochemical potentials, thereby providing a proper description of the electrons in the system. Finally, another feature which makes HP-DFT a one-of-a-kind formalism is its capability to accommodate a multiterminal setup. While exploring this aspect is beyond the scope of this manuscript, any number of probe sets with different electrochemical potentials could potentially be employed. As the focus of this paper primarily centered on benchmarking the formalism, our efforts were directed toward studying systems with a two-terminal setup for simplicity.

**Figure 1:**
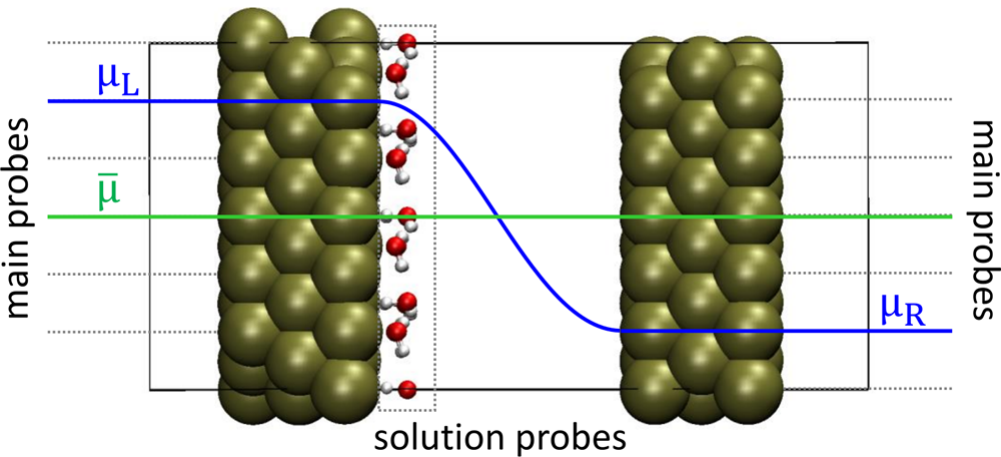
Representation of the parallel-plate capacitor models for HP-DFT calculations: two sets of main probes are coupled to the outer layers of each plate imposing μ_*L*_ and μ_*R*_ while solution probes are coupled to the atoms of the H_2_O molecules.

Overall, the key feature of HP-DFT is its efficiency. The integration of the Hairy-Probes formalism into the DFT code was easily achieved, requiring only a modification in the way the occupation numbers are calculated. As this is the sole deviation from the standard SCF cycle, the majority of the necessary variables are readily computed and supplied by the existing DFT code infrastructure. Consequently, the use of the HP-DFT formalism in calculations did not exhibit a significant deceleration of the SCF cycle in comparison to conventional DFT algorithms. Further information regarding the code’s efficiency is available in Figure S1 of the Supporting Material. The HP-DFT modules are available online at the ICL Nano Electrochemistry Group’s wiki.

In this section, we demonstrate the capability of the HP-DFT methodology to capture the properties of parallel-plate capacitor models, establishing its suitability to describe open-boundaries systems at fixed electrochemical potential difference.

First, we show that the imposed Δμ is reproduced and kept constant during the simulation. We applied an electrochemical potential difference between the two plates; values of Δμ = 1 eV and Δμ = 4 eV were considered. The average Hartree potential energy profile along the direction perpendicular to surfaces (shown in Figure 2) reveals the existence of two local Fermi-like levels corresponding to Δμ_*L*_ and Δμ_*R*_. The Projected Density of States (PDOS) on the left and right plate (see Figure 2) also shows a shift by |Δμ_*L*_| toward lower energies and by |Δμ_*R*_| toward higher energies respectively (μ̅ is set to zero in both cases).

**Figure 2:**
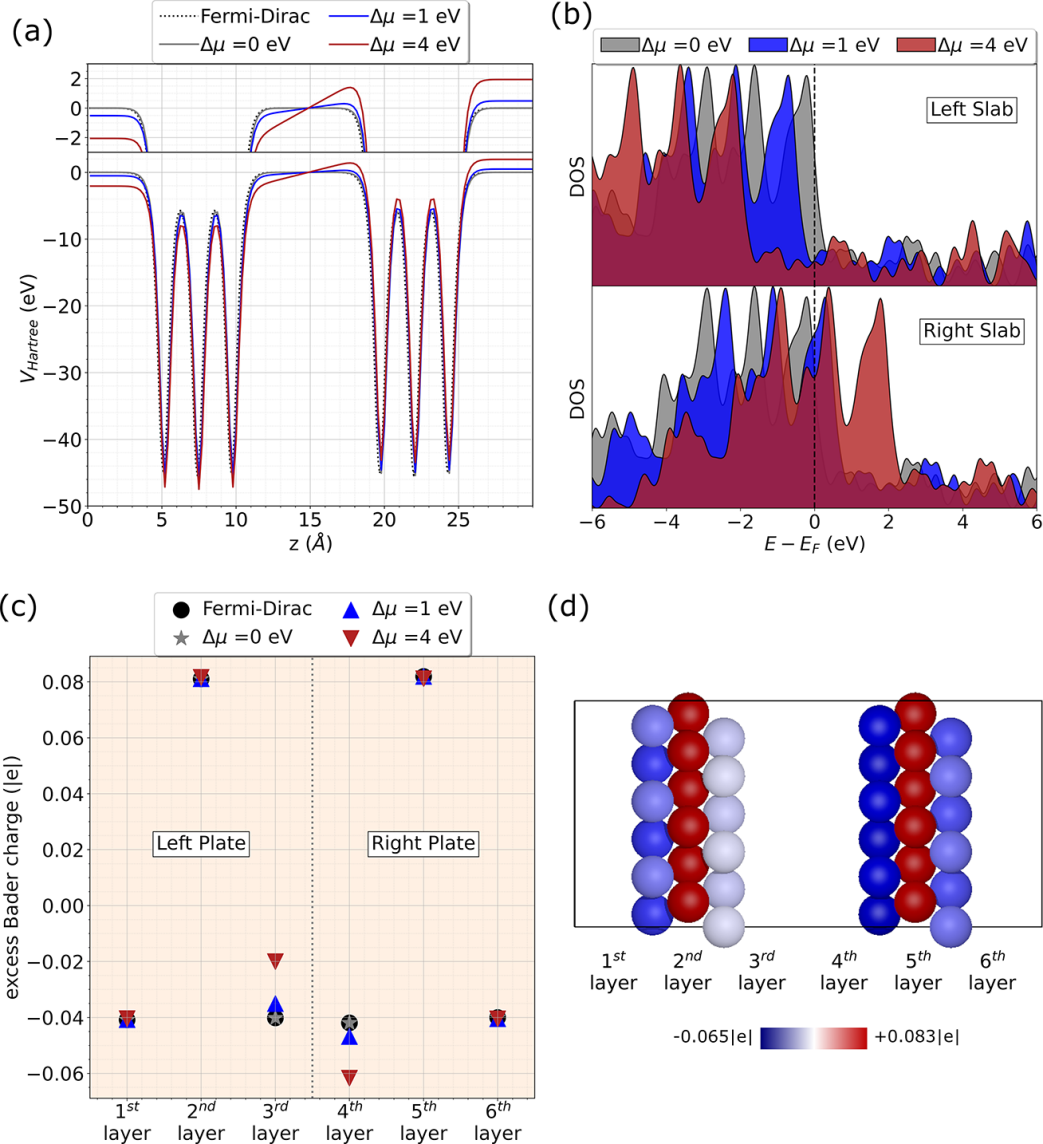
Pt(111)(6 × 6 × 3) system: (a) Average Hartree potential along the direction perpendicular to the surfaces (μ̅ is set to 0); (b) PDOS of the atoms of the plates’ inner layers (μ̅ is set to 0); (c) Average Bader charge per atom in each layer of the plates; (d) Bader charges for the Pt(111)(6 × 6 × 3) at Δμ = 4 eV.

Subsequently, we studied the charge distribution in the systems using Bader analysis.^47^Figure 2 shows the average excess Bader charge per atom (defined as the difference between the calculated Bader charge and the atom valence) in each layer of the plates. When Δμ = 0 eV the total excess Bader charge on both slabs (obtained from the sum of the excess Bader charge on each atom) goes to zero (within ±1^–5^ |*e*|). When Δμ ≠ 0 eV, the total excess Bader charge becomes negative on one slab and positive on the other. As can be observed in the figure, such variation in charge only occurs on the internal surfaces of the capacitor (3rd and fourth layers) as expected. Due to the high electronegativity of Pt, the atoms on the surfaces are negatively charged even at Δμ = 0 eV. Therefore, when Δμ ≠ 0 eV the average charge per atom on the internal surfaces will become “more negative” or “more positive” with respect to the zero bias case. To compensate, the charge of the subsurface (2nd and fifth layers in our 3-layer slabs), will have a positive charge. Figure (S2e) in the Supporting Material shows the average Bader charge per atom in each layer of the plates for the Pt(111)(2 × 2 × 5) system. It can be observed that in the 5-layer plates, while the subsurfaces (2nd and fourth layers for the left plate, and seventh and ninth layers for the right plate) are still positively charged, the charge tends zero in the middle of the slab (3rd and sixth layers). This observation indicates a realistic descrciption of the metallic slabs. The overall charge on the slabs for the Pt(111)(6 × 6 × 3) system is given in Table 1.

**Table 1:** Bader Charges, *q* (μC), and Capacitance, *C* (μF), at Different Values of Applied Δμ for Each Model System^a^

	**Pt(111)(2 × 2×3)**		**Pt(111)(2 × 2×5)**		**Pt(111)(6 × 6×3)**	
Δμ (eV)	*q* (μC)	*C* (μF)	*q* (μC)	*C* (μF)	*q* (μC)	*C* (μF)
1.00	3.37 · 10^–15^	3.37 · 10^–15^	3.37 · 10^–15^	3.37 · 10^–15^	3.03 · 10^–14^	3.03 · 10^–14^
4.00	1.35 · 10^–14^	3.37 · 10^–15^	1.34 · 10^–14^	3.36 · 10^–15^	1.21 · 10^–13^	3.03 · 10^–14^
Theoretical^a^	*C*_(2×2)_ = 3.34 · 10^–15^ μF				*C*_(6×6)_ = 3.01 · 10^–14^ μF	

a Values of capacitance calculated using the parallel-plate capacitor formula.

Finally, we calculated the capacitance for each of our models by evaluating *C* = *Q*/Δμ, where *Q* is the overall charge present on the plates. The values of capacitance thus calculated, reported in Table 1, are in good agreement with those calculated using the well-known formula *C* = ϵ_0_ × (*A*/*d*) (where ϵ_0_ is the vacuum permittivity, *A* is the plates’ surface area and *d* is their separation). Our results also confirm that the capacitance only depends on surface geometry, yielding the same value of capacitance regardless of the applied Δμ or slab thickness, while it varies when the surface area of the plates changes. Overall the capacitance per surface area calculated from the slab charge is 1.23 μF/cm^2^ and that calculated with the parallel plate capacitor formula is 1.22 μF/cm^2^.

Now we analyze the response of a simple water bilayer/platinum interface to the application of an electrochemical potential and we show that the HP-DFT methodology manages to correctly reproduce key behaviors of water adsorption without the need for large systems and computationally costly calculations.

Although water adsorption is not an electrochemical step, as it does not involve an electron-coupled-proton transfer process, we find that the surface polarization induced by the applied potential has a significant impact on water coverage, structure and adsorption energy. This is in contrast with the Nørskov assumption that only electrochemical steps in a reaction respond to changes in the electrode potential, while chemical steps are unaffected.^5^ Moreover, we show that the ideal water bilayer model does not capture the adsorption and coverage behavior of water over electrochemical interfaces under bias.

We studied water coverage and charge redistribution at Δμ = −4 eV, – 1 eV, 0 eV, + 1 eV and +4 eV. The EC potential difference is still applied such as Δμ_*L*_ = −Δμ_*R*_. In this, negative values of Δμ represent negative values Δμ_*L*_ (i.e., for Δμ = −4 eV, Δμ_*L*_ = −2 eV and Δμ_*R*_ = +2 eV). To this end, we calculated the adsorption energies as a function of Δμ for a series of structures with different coverages. The adsorption energies for the for insertion of a water bilayer into the charged capacitor, Δ*E*_*ads*_(Δμ), were calculated according to
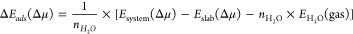
6where *E*_system_(Δμ) is the total energy of the capacitor+water system at Δμ, *E*_slab_(Δμ) is the energy of the slabs without water at Δμ,  is the energy of the molecule in gas phase and  is the number of adsorbed water molecules.

The results presented in Figure 3 show that at all EC potentials the H-down structures are more stable than the H-up configurations. The overall most stable configuration is the low coverage H-down bilayer, with only 8 chemisorbed H_2_O molecules. Generally, the low water coverage structures are the most stable ones for both configurations, with the exception of H-up at Δμ = −4 eV. In this case, one of the chemisorbed molecules in the first layer spontaneously desorbed from the surface during geometry optimization, effectively reducing water coverage (blue-colored molecule in Figure 4).

**Figure 3:**
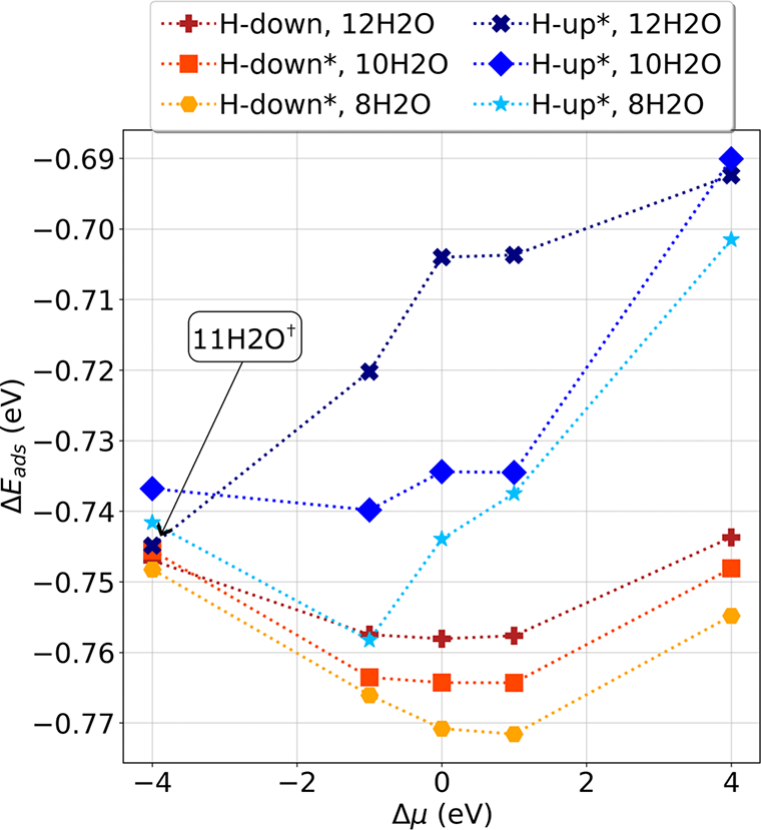
Adsorption energies for the water bilayer plotted as a function of the potential at different coverages: we compare both H-up and H-down configurations for cases with 12H_2_O, 10H_2_O, and 8H_2_O chemisorbed water molecules. * The initial bilayer configuration is H-up, but several molecules flip to H-down at negative Δμ. ^†^ One molecule desorbed from the 1st water layer.

**Figure 4:**
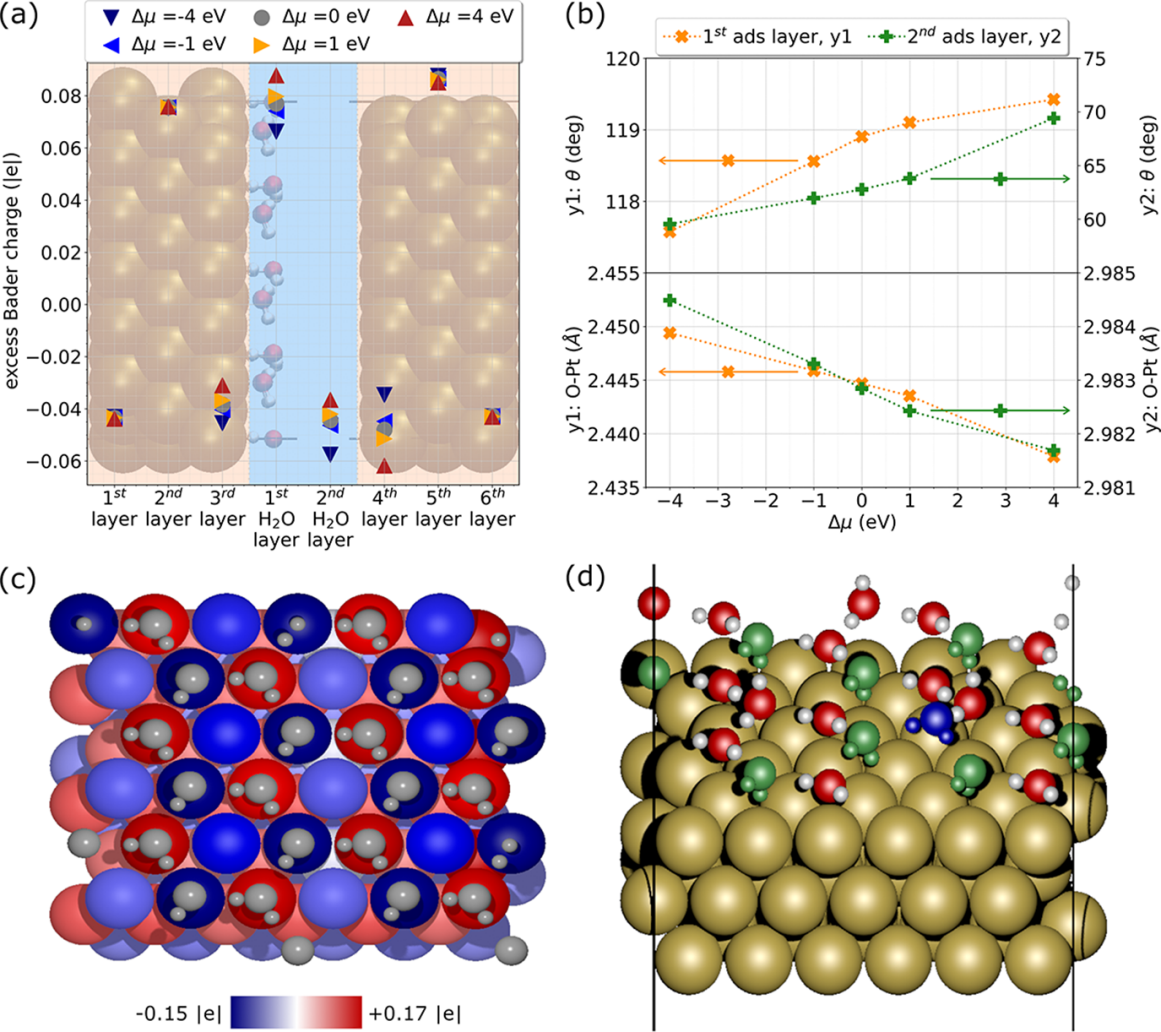
Pt(111)(6 × 6 × 3) system plus water bilayer: (a) average excess Bader charges of each atom of the plates and on each molecule of the bilayer, H-down configuration; (b) average θ and O–Pt distance for the molecules in the 1st (orange) and 2nd (green) adsorption layer, H-down configuration; (c) excess Bader charges on the left slab at Δμ = 4 eV, H-down configurations; (d) Pt(111)(6 × 6 × 3) system plus H-up water bilayer at Δμ = −4 eV: the green colored molecules flipped from H-up to H-down configuration while the blue colored one desorbed from the surface.

The stabilization of low water coverage structures can be explained in terms of the charge redistribution when the potential changes. Our data show that chemisorbed water molecules in the water bilayer transfer electrons to the metal surface, becoming positively charged, with an average charge between 0.06 and 0.09 |e|, depending on coverage and potential (see Table (S1) of the Supporting Material). The metal atoms to which they bind also have a net positive charge; the negative charge from the O–Pt bond spills over the neighboring metal surface atoms, causing them to become negatively charged, as shown in Figure 4. The presence of these negative Pt atoms destabilizes the ice-like bilayer structure even at Δμ = 0 eV (see Table (S2) of the Supporting Material). When the EC potential becomes more positive, the coverage of positively charged water molecules can increase but it saturates when additional negative charge transfer from water to the surface becomes unfeasible. At low EC potentials, the system cannot accommodate the positive bilayer, leading to the observed desorption.

Our methodology can be used for the modeling of electrochemical systems, where the constant potential of the electrode, is the potential difference between the working electrode and a reference level. In line with the work of Trasatti^48^ and Chen,^49−51^ this level can be represented by the vacuum point in front of the water layer or the bulk electrolyte level.To demonstrate this we have also evaluated the adsorption energy of the water bilayer on a half cell and, since we do not have bulk water in our system, we used as a reference the best evaluation for the vacuum level in our system. In this case, the agreement with AIMD studies is even more remarkable, as evidenced by the stabilization of a higher number of water molecules at high potentials due to the local charge redistribution induced by the application of an EC potential to the electrode. This observation, in fact, aligns with recent literature in showing that capacitive response of the interface to a positive EC potential is primarily driven by the increase in surface coverage of positively charged water molecules.^3,17,50^ This discussion is presented in more details in the Supporting Material.

The observations discussed in this section all align with recent AIMD studies^3,16,17,49,51−54^ which demonstrate that at zero bias the water coverage on the Pt electrode is lower than that modeled in the standard bilayer model. It is worth noting that these studies use exceptionally large and realistic models to simulate explicitly the polarization of the interfacial water layer when a potential is applied. However, this level of realism is achieved at the expense of substantial computational resources, while our results were obtained using significantly smaller systems.

Surface polarization is also compensated by structural changes in both water layers, as shown in Figure 4. As Δμ becomes more positive, the O–Pt distance decreases and θ (the angle between the normal to the surface and the water dipole) increases for both the first and second water layers. In the first layer, water molecules slightly reorient their H atoms toward or away from the surface at negative or positive potentials, respectively. The molecules of the second layer present the same behavior, but respond more freely to the change in surface charge and the observed reorientation is larger.

The analysis of interfacial charge redistribution also helps to explain water reorientation (see Figure 4). In the H-down structure, the attractive Coulombic interaction with the partially positive H stabilizes the negative charge on the Pt atoms. Indeed, the molecules of the second adsorption layer of the H-down configuration are closer to the surface (cf. Figure 4 and Figure (S2b) of the Supporting Material). Conversely, in the H-up structure the partially negative oxygen atoms of the second water layer point toward the surface, leading to Coulombic repulsion and a reduction in the negative charge on the Pt atom (lighter shades of blue in Table (S2) of the Supporting Material).

The trends discussed are clearly observable only for the more stable H-down configuration. However, consistent observations arise also from the analysis of the H-up system, although a clear geometrical reorganization trend is only seen for positive Δμ values, as can be observed from Figure (S2) and Table (S2) of the Supporting Material. This lack of monotonic trend in angles and distance is due to several molecules in the second layer flipping from an H-up to an H-down configuration at negative Δμ. For the full bilayer structures the number of molecules flipping at Δμ = −1 eV and −4 eV is, respectively, 2 and 9 out of 12 molecules in the second layer (green molecules in Figure 4). We observed this process for the low-coverage systems as well (Table (S2) of the Supporting Material). This so-called flip-flop process of the water molecules at charged interfaces has been experimentally documented^55,56^ and can be rationalized in terms of the response of the water molecule to the increasingly negative surface charge: the H-down configuration brings the positive part of the dipole closer to the increasingly negative surface and stabilizes the molecule.

We present a novel and efficient open-boundary formalism, HP-DFT, for modeling electrochemical systems. It combines the established Hairy Probes (HP) formalism with DFT in the canonical ensemble, offering computational efficiency comparable to standard DFT.

The HP-DFT formalism accurately reproduces parallel-plate capacitor models’ properties, demonstrating its suitability to describe open-boundary systems at fixed potential. The formalism successfully reproduces the imposed Δμ and keeps it constant during the simulations. Furthermore, the physics of the parallel-plate capacitor is correctly described and the capacitance obtained is in agreement with the theoretically evaluated one.

When applied to a water bilayer/platinum interface, HP-DFT successfully captures the key features of water adsorption on a charged metal plate, correctly describing charge redistribution and water reorientation. Our study finds that coverage, interface structure and adsorption energies are strongly influenced by the applied EC potential, despite water adsorption not being an electrochemical process. This is in contrast with Nørskov’s proposed model. Additionally, we find that the water bilayer does not accurately represent the adsorption behavior of a full water layer at electrochemical interfaces, as systems with lower coverage are energetically more stable even at zero bias. The results we found align with previous AIMD studies. Of course, one can expect that the presence of an extended bulk layer would affect the behavior of water at the interface. Nonetheless, despite starting as a simple proof of concept, our simplified model manages to qualitatively capture the expected behavior at a metal/water interface under bias, successfully accounting for local effects stemming from charge polarization. This highlights the advantages of using a methodology, HP-DFT, to directly control the electrochemical potential of the electrons in the electrode, as it can capture polarization response using small models and fine-tune the analysis of potential effects. Considering how lightweight the code itself is, the HP-DFT formalism proves an excellent candidate to model EIs by means of AIMD calculations with an open-boundary description of the electrons.

## Supporting Information Available

Additional detailed data for each of the systems discussed, including Tables and Figures. Additional analysis of a water monomer model on a charged Pt(111) surface, with corresponding discussion, tables and figures. Further discussion about the application of the methodology discussed for computational electrochemistry studies (PDF)
Transparent Peer Review report available (PDF)
