# Supplementary material for: Revealing Interface Polarization Effects on the Electrical Double Layer with Efficient Open Boundary Simulations under Potential Control
Source: J Phys Chem Lett. 2024 Apr 29;15(18):4872–9. doi: 10.1021/acs.jpclett.3c03615 (PMC11089570; doi:10.1021/acs.jpclett.3c03615)
Supplement: Supplementary file 1 — jz3c03615_si_001.pdf [file jz3c03615_si_001.pdf]

Revealing Interface Polarisation Effects on the Electrical Double Layer with Efficient Open Boundary Simulations under Potential Control

Margherita Buraschi,[*,†] Andrew P. Horsfield,[‡,¶] and Clotilde S. Cucinotta[*,†,¶]

†*Department of Chemistry, Imperial College London, White City Campus, London W12 0BZ, UK*

‡*Department of Materials, Imperial College London, South Kensington Campus, London SW7 2AZ, UK*

¶*Thomas Young Centre*

E-mail: m.buraschi20@imperial.ac.uk; c.cucinotta@imperial.ac.uk

Supporting Material

Code efficiency

Profiling of the code shows that the HP-DFT formalism did not result in a relevant slowdown of the SCF cycle compared to standard DFT algorithms. For example, a model such as the Pt(111)(6×6×3) plus water bilayer described in the paper consists of 216 Pt atoms and 24 water molecules for a total of 4080 valence electrons and 2096 occupied MO (3040 total MO, 8256 independent orbital functions). The average time per SCF cycle step for such a system was circa 4.4 s for standard DFT, while the HP-DFT calculation at $\Delta\mu = 0$ eV took around 5.7 s per cycle. For both calculations, the geometry was fully relaxed in 15 optimization steps with a similar number of SCF cycle steps. Even at $\Delta\mu = 1$ eV, the average time per SCF

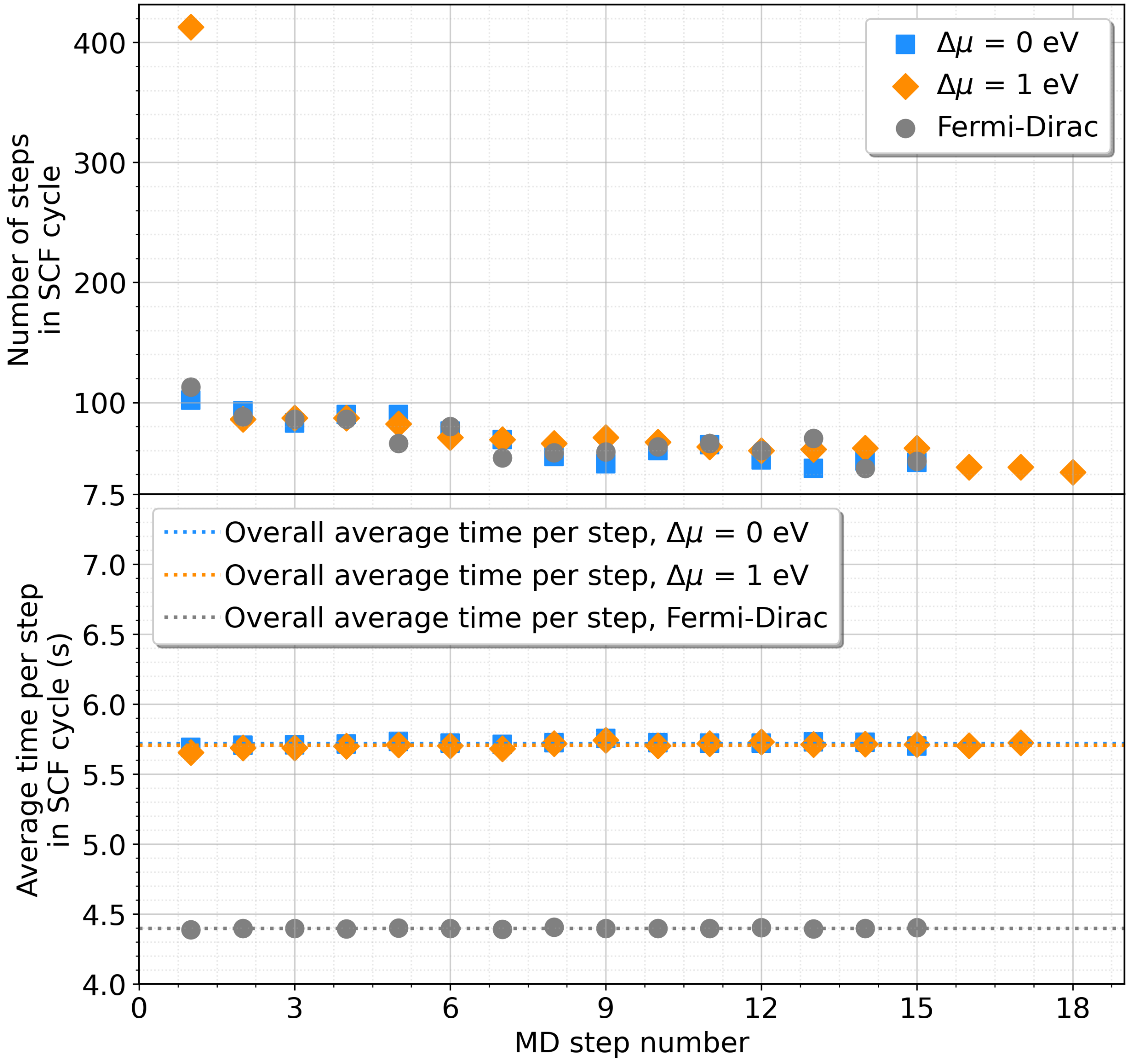

Figure S1: Top: number of steps in each SCF cycle. Bottom: average time per step in each SCF cycle.

cycle step was around 5.7 s. In this case, the geometry took 18 steps to fully relax. This is summarized in Figure (S1). This minimal time difference can be attributed to the fact that the necessary quantities to determine the HP-DFT occupation numbers, f_i, using Equation (3) of the paper are already computed by the underlying DFT code. As a result, the HP-DFT approach proves to be computationally efficient, making it particularly appealing to control and tune the electrochemical potential in a simulation. It is worth noting that this efficiency extends to applications in combination with *ab initio* molecular dynamics (AIMD) calculations; although ongoing preliminary tests show a mild slowdown in a system with a large number of molecules in solution, the average time per SCF cycle remains comparable to that of a standard AIMD calculation.

Slab description in capacitor models

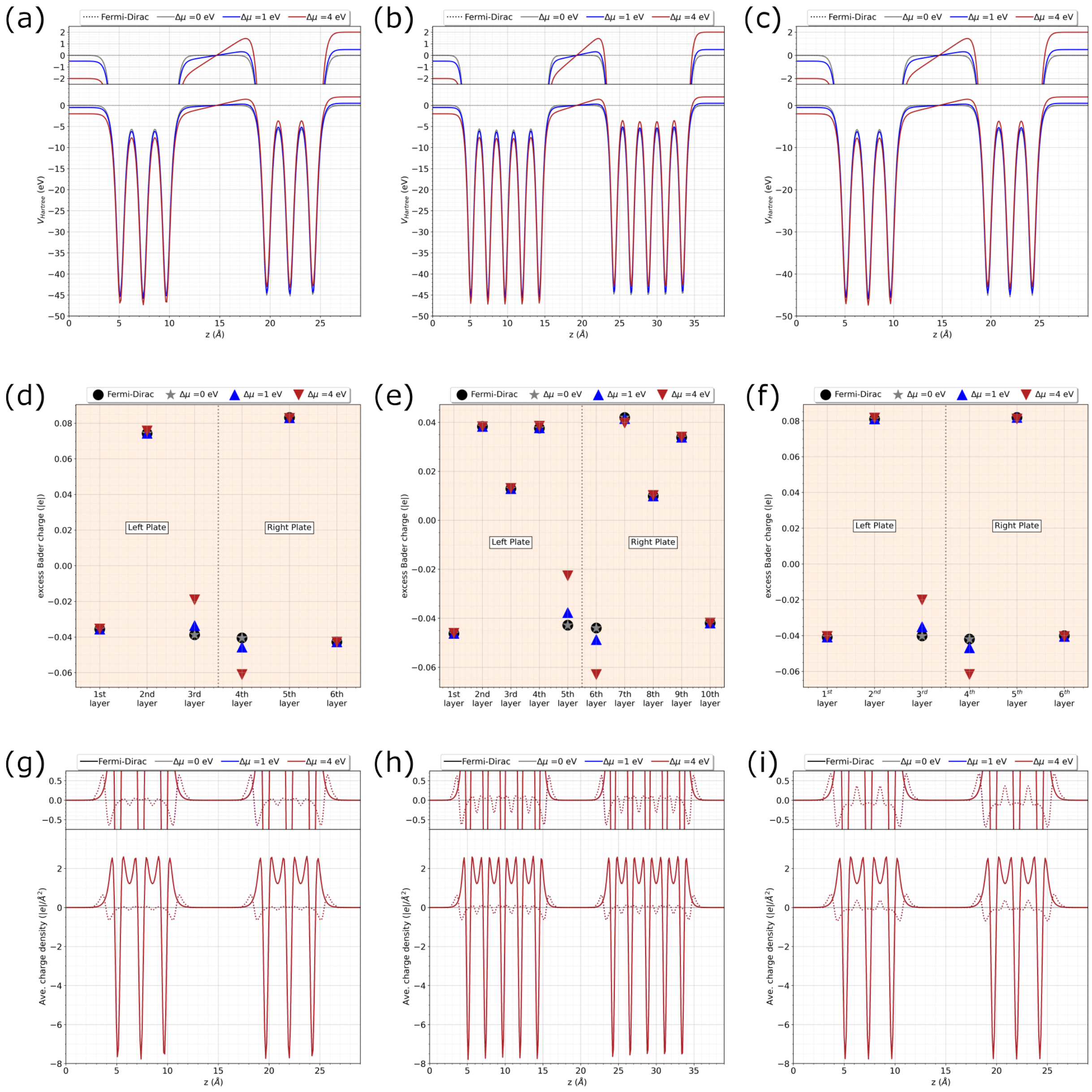

Figure S2: Parallel-plate capacitor models: average Hartree potential along the direction perpendicular to the surfaces ($\overline{\mu}$ is set to 0), average Bader charge per atom in each layer of the plates and average charge density along the direction perpendicular to the surfaces for Pt(111)(2×2×3) system (a), (d) and (g); Pt(111)(2×2×5) system (b), (e) and (h); Pt(111)(6×6×3) system (c), (f) and (i).

Figures (S2a), (S2b) and (S2c) show the average Hartree potential along the Z-axis for the Pt(111)(2×2×3), Pt(111)(2×2×5) and Pt(111)(6×6×3) systems, respectively. The local

Fermi levels of the left and right plates are identifiable in each model.

Figures (S2d) to (S2i) illustrate the charge distribution within the plates of each model. Due to the high electronegativity of Pt, Pt(111) surfaces are negatively charged[1–3] and, as a consequence, the subsurfaces are positively charged. The charge then should go to zero in the middle of the slab, where bulk conditions are achieved. In our 3-layer systems, Figures (S2d) and (S2f), the middle layers for both slabs (2nd and 5th layers) are also the subsurfaces to both surfaces. Consequently, they are positively charged to compensate the negative charge on the surface. In our 5-layer model, while the subsurfaces (2nd and 4th layers for the left plate, and 7th and 9th layers for the right plate) are still positively charged, the charge tends zero in the middle of the slab (3rd and 6th layers), as shown in Figure (S2e).

It is worth noting that the magnitude of the average charge per atom is a result of Bader analysis, which integrates over atomic volumes. Plotting the average total charge density distribution along Z, however, we can see that the total charge goes to zero in the middle of the metal slab for all systems, as shown in Figures (S2g), (S2h) and (S2i).

Single water molecule adsorbed on a charged surface

Initially, we performed a set of calculations with only a single water molecule adsorbed on both the internal surfaces of the Pt(111)(6×6×3) capacitor. We performed HP-DFT calculations at $\Delta\mu = 0, 1$ and 4 eV and we studied the response of the adsorbates to the surface charge redistribution induced by the application of a potential.

Figure (S3a) shows the charge distribution as a function of the applied $\Delta\mu$. As expected, the charge of the plates varies only on the internal surfaces of the capacitor; at the same time, the molecule adsorbed on the left surface becomes more positive while the one adsorbed on the right surface becomes more negative. The more positive the right surface becomes the more electronic charge H_2O donates to the electronegative Pt surface. Conversely, as the left electrode surface becomes more negatively charged, less electronic charge is donated to Pt; consequently, the molecule becomes less and less positively charged, going towards zero

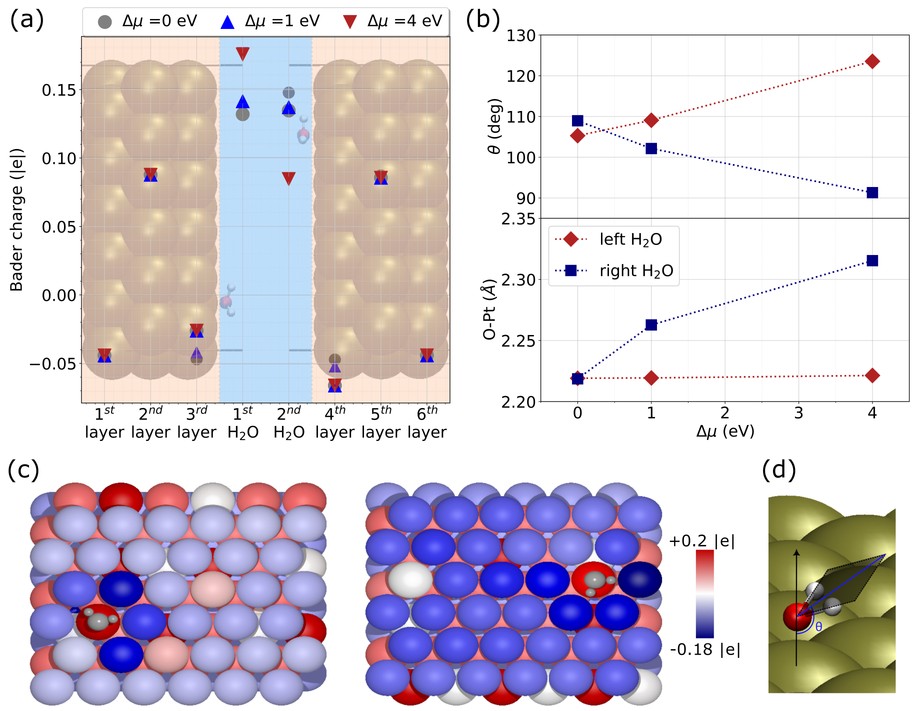

Figure S3: Pt(111)(6x6x3) system plus water molecule: (a) average excess Bader charges per atom in each layer of the plates and per molecule; (b) θ and O-Pt distance for the molecules adsorbed on the left (red) and on the right (blue) slab; (c) Excess Bader charges on the left and right surface; (d) definition of θ.

(the charge of the isolated molecule).

Figure (S3b) shows water re-orientation as a response to the varying surface charge. As $\Delta\mu$ increases, the O-Pt distance decreases for the molecule on the left surface while it increases for the molecule adsorbed on the right surface. At the same time, defining θ as the angle between the normal to the slab surface and the water dipole (shown in Figure (S3d)), we observe that its value increases on the left surface while it decreases on the right one. In other words, the water molecule adsorbed on the left slab rotates to point its oxygen towards and closer to the surface; the opposite happens on the right slab, where the water molecules rotate to move its oxygen away from the surface.

Figure (S3c) shows the charge distribution on both the left and right surface at $\Delta\mu = 4$ eV. In both cases, the Pt atom acting as the adsorption site carries a more positive charge while its direct neighbours carry, instead, a more negative charge (compared to the other surface

atoms). The electronegative platinum surface withdraws charge from the water's oxygen, resulting in a positively charged molecule. The electron charge originally on water is then transferred from the adsorption site to the surface atoms surrounding it, which become more negative.

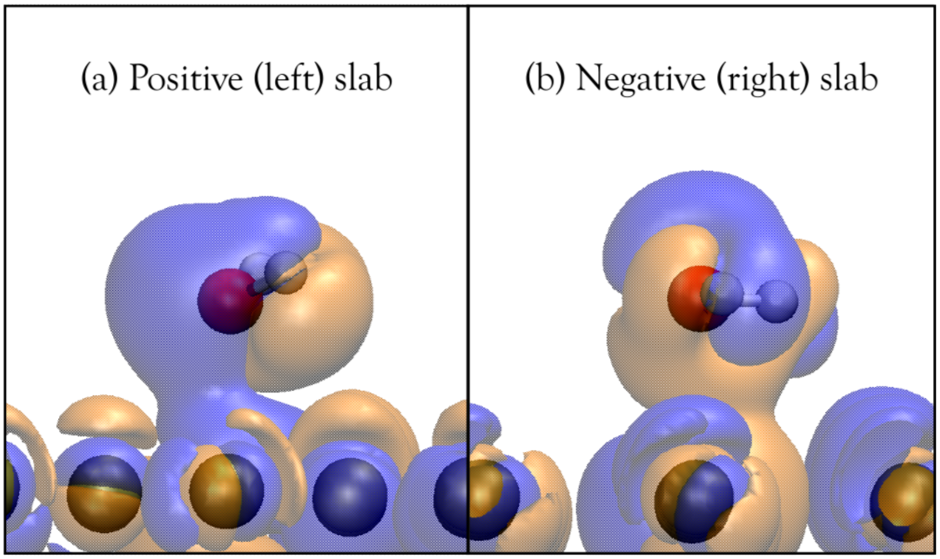

Figure S4: Electron density difference represented as isosurfaces between the system at $\Delta\mu = 4$ eV and the system at $\Delta\mu = 0$ eV: (a) molecule on the left plate, (b) molecule on the right plate. The blue and orange surfaces represent isolevels at -0.002 e/Å and 0.002 e/Å respectively (negative values represent electron accumulation).

Figure (S4) shows the electron density difference between the system at $\Delta\mu = 4$ eV and the system at $\Delta\mu = 0$ eV. An accumulation of electronic charge (indicated by the blue isosurface) between the water and the left plate, suggesting a stronger bond between the molecule and the surface. Conversely, a depletion of electronic charge (indicated by the orange isosurface) between the water and the right plate indicates a weakening of the molecule-surface bond. Both observations are in agreement with the data obtained from the Bader and geometry analysis of the system.

Excess Bader charges on Pt(111)(6×6×3) bilayer system

Table S1: Total Bader charge on the left and right metallic plates and average Bader charge per molecule in the 1st and 2nd adsorption layer for the H-down (top) and H-up (bottom) configuration.

**H-down configuration**				
$\Delta\mu$ (eV)	Total charge on plate (|e|)		Average charge on molecule (|e|)	
	Left plate	Right plate	1st ads. layer	2nd ads. layer
-4.00	-0.476	0.365	0.066	-0.058
-1.00	-0.294	-0.029	0.074	-0.046
0.00	-0.225	-0.161	0.077	-0.044
1.00	-0.161	-0.293	0.08	-0.042
4.00	0.042	-0.707	0.09	-0.036
**H-up configuration**				
$\Delta\mu$ (eV)	Total charge on plate (|e|)		Average charge on molecule (|e|)	
	Left plate	Right plate	1st ads. layer	2nd ads. layer
-4.00	-0.539	0.312	0.061	-0.034
-1.00	-0.359	-0.259	0.064	-0.009
0.00	-0.3	-0.459	0.059	-0.001
1.00	-0.219	-0.595	0.062	0.006
4.00	0.013	-0.998	0.07	0.011

Table (S1) reports the total Bader charge on the metallic plates as well as the average Bader charge per molecule in the 1st and 2nd adsorption layer. As $\Delta\mu$ goes from -4 eV to 4 eV, we can observe the surface charge on the left plate becoming more positive; at the same time, the average charge on the molecules of both the 1st and the 2nd adsorption layer becomes more positive. This indicates that more charge is passed from the water to the surface as the plate becomes more positively charged. Noticeably, the molecules in the 2nd adsorption layer of the H-up configuration have a much smaller charge than their counterpart in the H-down configuration: this observation suggests that these waters are less bonded to the

surface and are stabilized mainly by their hydrogen bonds with the molecules in the 1st. The overall charge of the left plate + water bilayer is compensated by the surface charge on the right plate (the counter electrode) which, accordingly, becomes negative with increasing $\Delta\mu$.

Table S2: Excess Bader charges on the left plate of the Pt(111)(6×6×3) plus H-down and H-up bilayer; both the full bilayer and the low coverage systems are shown. The green dotted circles in the H-up systems indicate the molecules in the 2nd adsorption layer which flip from an H-up to and H-down configuration as $\Delta\mu$ becomes more negative. The yellow dot-dashed circles in both the H-down and the H-up systems indicate the molecules in the 2nd adsorption layer which bring their dipole almost parallel to the surface.

H-down configuration

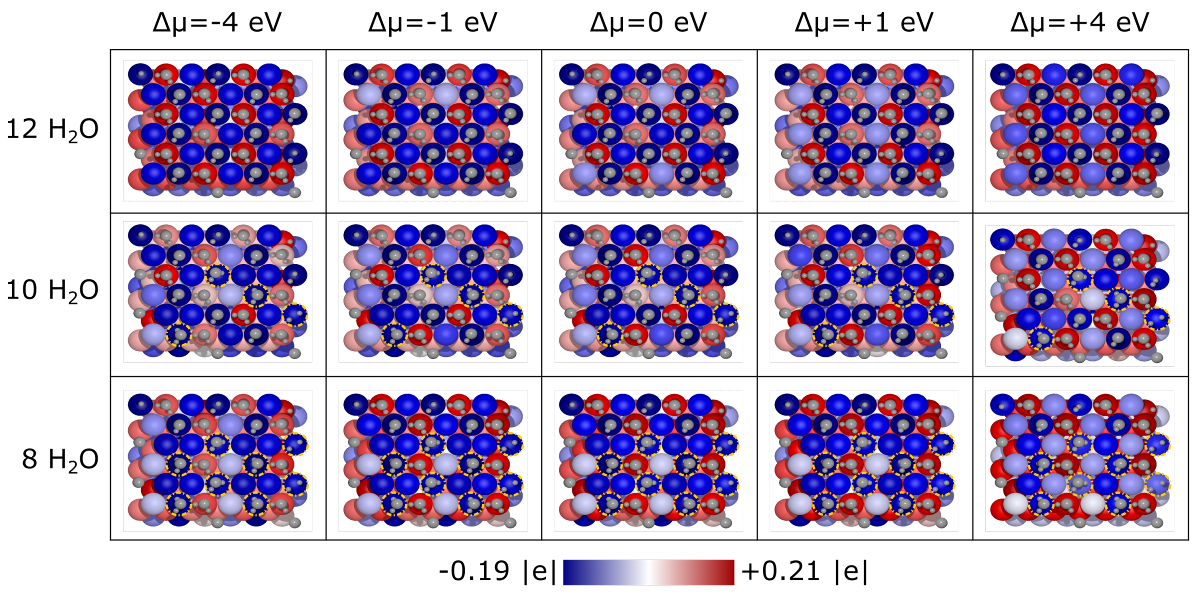

H-up configuration

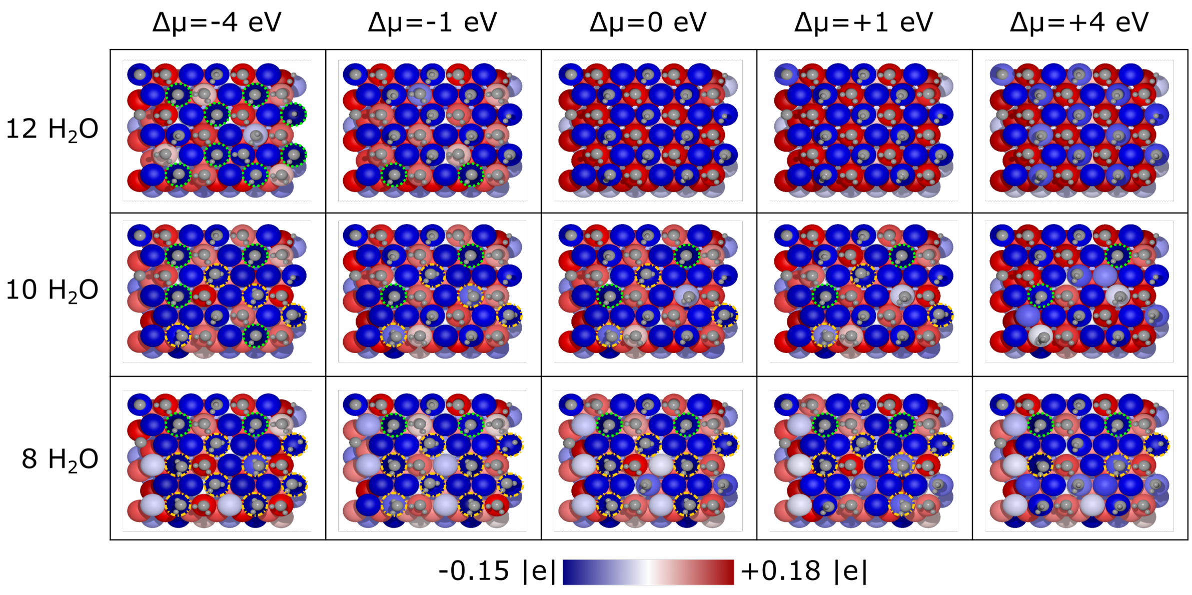

Table (S2) shows the excess Bader charges on the left plate of the Pt(111)(6×6×3) plus bilayer systems in the H-down and H-up configurations. Both the full bilayer and the low coverage systems are shown. As a result of the removal of some molecules from the 1st layer, some molecules in the 2nd are not stabilized by the hydrogen bonds and adopt a configuration that neither H-up nor H-down: their dipole becomes almost parallel to the surface, with both hydrogens pointing slightly down towards the plate. In the H-down system, the number of molecules in the 2nd layer which become almost parallel to the surface does not seem to be particularly influenced by the value of $\Delta\mu$ (yellow dot-dashed circles in Table (S2), H-down configuration). In the H-up system, on the other hand, we observe an interesting trend. When the coverage is high, there is a progressively higher number of 2nd layer molecules flipping from an H-up to an H-down configuration as $\Delta\mu$ becomes more negative (green dotted circles in Table (S2), H-up configuration). In the low coverage systems, however, when the potential is low the response to polarisation occurs mostly through an increasing number of molecules in the 2nd layer re-orienting themselves in the almost parallel configuration (yellow dot-dashed circles in Table (S2), H-up configuration).

Pt(111)(6×6×3) bilayer system in the H-up configuration

Figure (S5a) shows the charge distribution as a function of the applied $\Delta\mu$ while Figure (S5b) shows water re-orientation in the 1st (orange) and 2nd (green) adsorption layer as a response to the varying surface charge. The trends discussed in the paper for charge distribution and water re-orientation are clearly visible only for positive values of $\Delta\mu$ while the data for negative values of $\Delta\mu$ is more disordered (as can also be observed in Table (S1)). This happens because several molecules of the 2nd adsorption layer flip from an H-up to an H-down configuration.

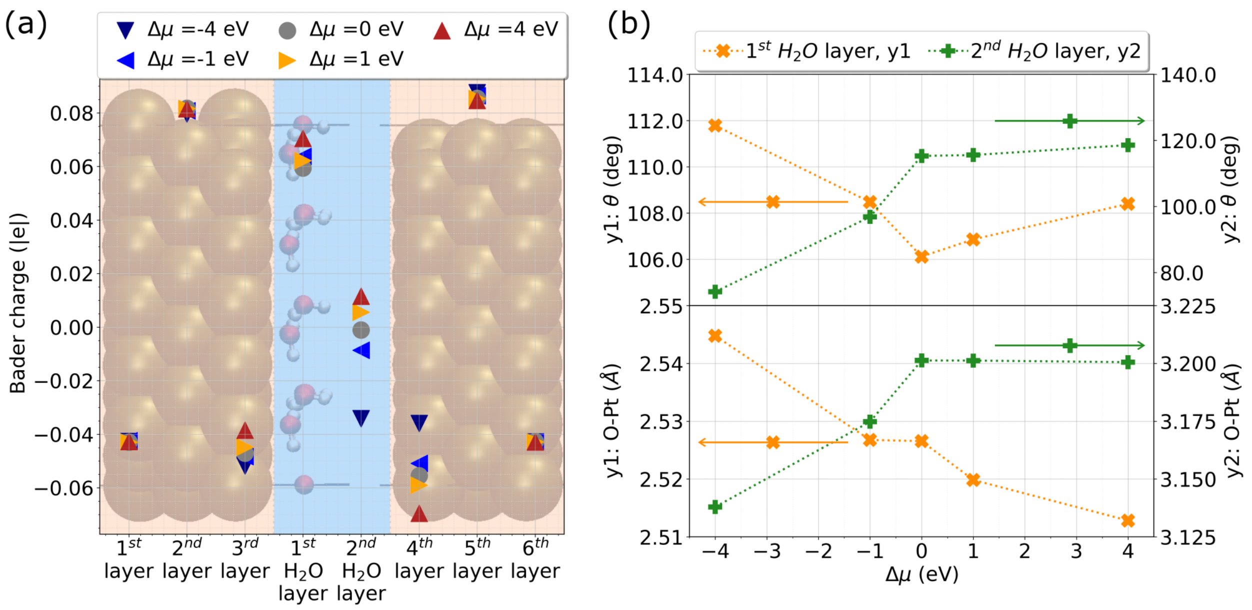

Figure S5: Pt(111)(6×6×3) system plus water H-up bilayer: (a) average excess Bader charges of each atom of the plates and on each molecule of the bilayer; (b) average θ and O-Pt distance for the molecules in the 1st (orange) and 2nd (green) adsorption layer.

Application to Electrochemistry

We would like to address how this methodology can be used for electrochemical applications. In EC studies the electrode potential needs to be explicitly included in the evaluation of adsorption energies. Consequently, a sensible reference level needs to be established. This can be done, for example, in situations where an extended bulk region is included in the simulation, where the potential of the bulk water could serve as the reference level. Such a level, however, does not exist in our systems, as there is not enough water to screen the dipole. Consequently, we have evaluated the adsorption energy of the water layer on a half cell, using as a reference the best evaluation for the vacuum level in our system. For the sake of this discussion, we will take the Hartree potential of in the middle of the vacuum region (where the average Hartree potential profiles for all the systems cross) as the reference value to calculate the "work functions" of the left and right electrode. Indeed, if we applied a dipolar correction in the vacuum region to cancel out the field, the resulting vacuum level would correspond to the value of the reference level indicated in Figure (S6b). These "work

functions" therefore are calculated as:

$$W_L = V_{middle} - \mu_L \tag{1}$$

and

$$W_R = V_{middle} - \mu_R \tag{2}$$

where V_{middle} in the Hartree potential of in the middle of the vacuum region and μ_L and μ_R are the local Fermi levels on the left and right plate respectively. The inclusion of these terms in the equation for the adsorption energies then becomes:

$$\begin{aligned}\Delta E_{ads}(\Delta\mu) =& \frac{1}{n_{H_2O}} \times \{E_{\text{system}}(\Delta\mu) - E_{\text{slab}}(\Delta\mu) - n_{\text{H}_2\text{O}} \times E_{\text{H}_2\text{O}}(\text{gas}) \\ & + [q_L(\Delta\mu) \times W_L(\Delta\mu) + q_R(\Delta\mu) \times W_R(\Delta\mu)]\}\end{aligned} \tag{3}$$

where $W_L(\Delta\mu)$ and $W_R(\Delta\mu)$ are the "work functions" of the left and right electrode at $\Delta\mu$ and $q_L(\Delta\mu)$ and $q_R(\Delta\mu)$ are the excess Bader charges on the left and right plate at $\Delta\mu$. The resulting adsorption energies are reported in the following plot: The effects of including the term between square brackets in Equation (3) are presented in Figure (S6a). The data in Figure (S6a) indicates that the low coverage H-down structures are still the most stable ones for values of $\Delta\mu$ between -1 eV and +1 eV. At $\Delta\mu = 4$ eV, however, the full coverage H-down water bilayer becomes the most stable structure. At $\Delta\mu = -4$ eV, instead, the full coverage H-up bilayer structure is the most stable one. In this system, most of the molecules in the 2nd adsorption layer flipped from an H-up to an H-down configuration, stabilizing the bilayer. This is also the structure where one of the chemisorbed molecules in the 1st layer spontaneously desorbs from the surface during geometry optimization. Such reduction in water coverage further stabilizes the bilayer making it more stable than its full coverage H-down counterpart. This observation aligns with recent literature in showing that capacitive response of the interface to a positive EC potential is primarily driven by the increase in

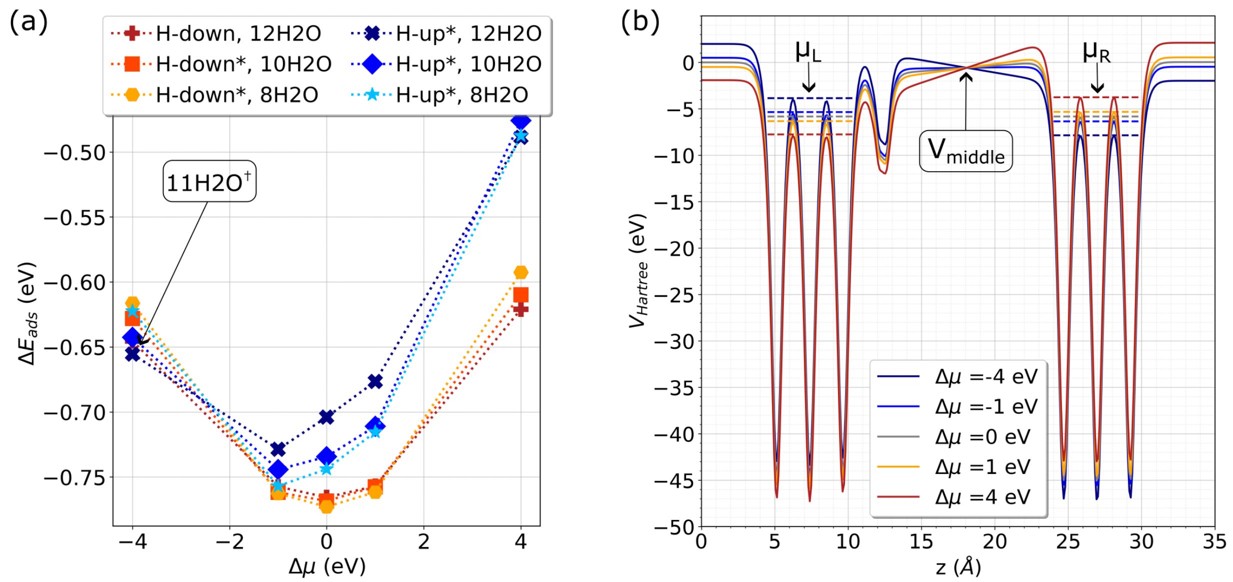

Figure S6: (a) Adsorption energies for the water bilayer plotted as a function of the potential at different coverages as calculated using Equtaion (3): we compare both H-up and H-down configurations for cases with 12H_2O, 10H_2O, and 8H_2O chemisorbed water molecules. * The initial bilayer configuration is H-up, but several molecules flip to H-down at negative $\Delta\mu$. † One molecule desorbed from the 1st water layer; (b) Average Hartree potential along the direction perpendicular to the surfaces (solid lines) and local Fermi levels (dashed lines) for the Pt(111)(6×6×3) plus water bilayer system ($\overline{\mu}$ is set to 0).

surface coverage of positively charged water molecules.[1,3,4]

In a system with a well defined and sensible reference level, the same equations (1) to (3) can be applied using such level instead of V_{middle}.

References

(1) Khatib, R.; Kumar, A.; Sanvito, S.; Sulpizi, M.; Cucinotta, C. S. The nanoscale structure of the Pt-water double layer under bias revealed. *Electrochim. Acta* **2021**, *391*, 138875.

(2) Surendralal, S.; Todorova, M.; Neugebauer, J. Impact of water coadsorption on the electrode potential of H-Pt(1 1 1)-liquid water interfaces. *Phys. Rev. Lett.* **2021**, *126*, 166802.

(3) Darby, M. T.; Cucinotta, C. S. The role of water at electrified metal-water interfaces unravelled from first principles. *Curr. Opin. Electrochem.* **2022**, *36*, 101118.

(4) Le, J.-B.; Fan, Q.-Y.; Li, J.-Q.; Cheng, J. Molecular origin of negative component of Helmholtz capacitance at electrified Pt(111)/water interface. *Sci. Adv.* **2020**, *6*, eabb1219.
