# Supplementary material for: Revealing Interface Polarization Effects on the Electrical Double Layer with Efficient Open Boundary Simulations under Potential Control
Source: J Phys Chem Lett. 2024 Apr 29;15(18):4872–9. doi: 10.1021/acs.jpclett.3c03615 (PMC11089570; doi:10.1021/acs.jpclett.3c03615)
Supplement: Supplementary file 2 — jz3c03615_si_002.pdf [file jz3c03615_si_002.pdf]

jz-2023-03615x.R1

Name: Peer Review Information for "Revealing Interface Polarisation Effects on the Electrical Double Layer with Efficient Open Boundary Simulations under Potential Control"

First Round of Reviewer Comments

Reviewer: 1

Comments to the Author

This manuscript investigates the behavior and properties of interface water molecules under different potentials with a new constant potential DFT algorithm (i.e., HP-DFT). This new algorithm is interesting and promising. However, the interface model used in the manuscript seems to have serious issues, making the reliability of the conclusions questionable.

1. The manuscript did not clearly outline the advantages of the HP-DFT algorithm compared with other constant potential algorithms.
2. In the "HP-DFT formalism" section, the authors mention a coupling strength parameter Γ_p, but this parameter is not observed in subsequent formulas. It has also not been clarified how to calculate the average electron electrochemical potential $\bar{\mu}$.

   Additionally, the authors applied μ_L and μ_R based on the assumption of $\Delta\mu_L = -\Delta\mu_R$. However, this assumption may not be appropriate, as the simulation model is not symmetrical and water molecules are present only on one side of the electrode.
3. The interface model used in this manuscript contains a small number of water molecules only at one side electrode, while different electrochemical potentials are applied to both electrodes. In this model, water molecules may be unable to screen the excess charges on both electrodes, which could lead to serious issues with periodic boundary conditions and result in unreliable simulation results. To address this issue, the author should consider an implicit solvent model or dipole correction, or widen the electrode spacing, incorporating a sufficient number of water molecules, and conducting AIMD calculations.
4. The distribution of excess charges appears to be inaccurate. In Figure 2, the atoms in the middle layers (i.e., the 2nd and 5th layers) carry positive charges on both positive and negative electrodes, and the amount of those charges is higher than that of the inner layers (i.e., the 3rd and 4th layers). This contradicts some existing theories and computational results (*Nat Commun* 13, 174, 2022;

Nat Commun 9, 716, 2018; *Phys. Rev. B* 7, 3541, 1973), where the excess charges in the electrodes are primarily distributed in the inner layer, with fewer excess charges in the remaining layers.

5. The electrochemical stability window of water is 1.23 V, but the electrochemical potential range calculated in the manuscript is -4 eV to 4 eV, far exceeding the electrochemical window of water molecules. At such high voltages, water molecules should have already undergone reactions.

Reviewer: 2

Comments to the Author

The study conducted by Buraschi and colleagues explores the polarization effects of the electric double layer at the Pt(111)/water interface. The main novelty of this work is integrating a constant potential method originally developed by Zauchner and co-workers ((PRB, 2018, 97, 045116)) into CP2K. That method facilitates the examination of electrochemical interfaces at predetermined chemical potentials, a feature of significant interest in fields such as electrocatalysis, corrosion, and electroplating. Despite the attractiveness of the constant potential method for modeling electrochemical interfaces, the manuscript does not clearly delineate the main advantages of the used method over existing constant-potential methods, such as those proposed by Bonnet, PRL, 2012, 109, 266101 and Bouzid, JPCL, 2018, 9, 1880. Furthermore, the investigation into water orientation and adsorption energy in response to electrode potential, utilizing a simplified water bilayer model, does not convincingly reveal novel electrochemical insights. Consequently, this work appears to be more methodologically focused, suggesting its suitability for a journal with a technical emphasis.

Minor Comments for Clarification and Improvement:

1) In this work, the terminology “constant potential” is used to describe the potential difference between a working electrode and a counter electrode. However, it is not the case of electrochemistry, in which the “constant potential” should be the potential difference between a working electrode and a reference electrode. I am not convinced how this method can be used for studying electrocatalysis at the so-called constant potential condition.

2) The calculation of water adsorption energy, as outlined in Equation (6), raises questions. Typically, the reference state for water would be its gaseous form, making the inclusion of the last term in Equation (6) questionable. A revision or justification for this term would be beneficial.

3) To better elucidate water polarization at charged surfaces, considering a water monomer model might offer a more straightforward analysis.

4) In presenting calculated capacitance values in Table 1, adopting the unit “uF/cm2” would facilitate easier comparison with both experimental and theoretical studies in the field.

5) The discrepancy in slab separation distances—10 angstroms for some models and 20 angstroms for others—within the method section warrants explanation. Clarifying the rationale behind these choices would aid in understanding the modeling approach.

Reviewer: 3

Comments to the Author

The manuscript by Buraschi, Horsfield and Cucinotta discusses the implementation of the hairy probes formalism, in which each atom at an interface of interest is connected to a lead imposing a desired potential, into the CP2K package. This approach is then applied to study properties of a water bi-layer/Pt(111) interface. The work is interesting, but there are limitations I elaborate on below, which make the manuscript unsuitable for publication in its present form. After these have been addressed, the manuscript should be re-evaluated.

1) The authors write that the leads are typically coupled only to atoms in the contact region. Obviously, this applies to the electrode and is needed to achieve a potential drop at the interface. They also mention the implementation of “solutions probes” needed to avoid a “nonphysical situation where molecules in solution have no electrons”. Do I understand correctly that this “solution leads” couple to water molecules, so that this coupling leads to a surplus/deficiency of electrons on the water molecules? What are the charges on the water molecules?

2) Some of the applied potentials are rather high and I would expect reactions to occur. Yet the shown effects are molecule desorption or reorientation. It is not clear to me, whether reactions, for example water dissociation, can occur. How will these be affected by the “solution leads”?

3) If I understand the set-up correctly, the investigated interface consists of a Pt(111) slab in contact with two water layers, which in turn are connected to a vacuum region. What about a bulk water region? The properties of an adsorbed water bi-layer in vacuum are not necessarily the same as the properties of an adsorbed water bi-layer in contact with water. Yet the shown results are often compared to calculations in which an extended water region is present. Is such a comparison applicable? Are the comparisons of timing of calculations using an extended bulk water region fare? Obviously any calculations which does not have to take care of equilibrating a bulk water region will be faster.

4) I find the discussion of “The response of a water bilayer to the charge of electrode potential” to be a bit selective on the comparison to available literature and selection of which articles to cite. The understanding of electrochemical interfaces has evolved since the cited 2004 article and people are aware that changes in the electrode potential affect the polarisation, orientation and adsorption/desorption behaviour of water molecules at the interface, even if electron transfer reactions have not yet occurred.

5) In the discussion of the set-up the authors mentioned two numbers for the separation between the Pt slabs - 10 Ang and 20 Ang. It is not clear to me, whether the separation of 20 Ang applies to all the models containing water or only to the ones with a reduced number of water molecules. It is also not clear why it was necessary to change the separation length.

6) Why is an entropy term included only for the gas-phase molecule? What about the water molecules at the interface? What about entropic contribution by the bi-layer water molecules? Surely this will be lower, but not necessarily negligible. Do the authors have an estimate about their magnitude and whether neglecting them is justified?

7) I have difficulties with the formulation “the potential increases” (p.11). I assume the authors mean “becomes more positive/negative”?

8) What does a “full coverage water bilayer” mean – a 12 water molecules structure in the first and 12 water molecules in the second layer? What is the meaning of “low water coverage structures”? Please be more precise.

9) I believe that a citation is missing on p.12 when the authors say that their “results also align with recent literature”.

10) What is the meaning of E_h?

11) Please be more specific when writing that the “HP-DFT formalism in calculations did not exhibit a significant deceleration of the SCF cycle.” Some numbers were provided in the SI comparing timings for one cycle. What is not clear to me is, how long does the overall equilibration of the system take. After all, one needs to equilibrate the various leads. How efficient the overall performance is, would likely very much depend on the implementation and used algorithm.

12) While I can guess which DOS in Fig. 2b relates to the left and which to the right lead, it would be helpful to also write this explicitly in the figure.

13) It would be helpful to incorporate the labels of the graphs in Fig. 4b into the figure.

14) How are distances measured? Do the authors use the centre of mass of a water molecule or maybe the oxygen in a water molecule?

Author's Response to Peer Review Comments:

Dear Editor,

Thank you for considering our manuscript for publication in “The Journal of Physical Chemistry Letters” and for forwarding the comments of the referees. We have carefully reviewed their comments and suggestions, and we are pleased to submit our detailed responses addressing all technical and analytical issues raised.

Attached, you will find our point-by-point response to the referees' comments (highlighted in yellow), along with a list of the additional changes made to the manuscript in light of their feedback, with additions marked in blue for the main text and in red for the supplementary material.

We have included two versions of the revised Manuscript and Supplementary Information:

The documents " main_reviewed.pdf " and " Supporting_reviewed.pdf " highlight the changes made compared to the previous version of the manuscript. They are uploaded as part of the manuscript zip file. Please note that references to line numbers, tables, figures, and equations correspond to these revised documents.

Additionally, we have provided polished versions of both the manuscript and the Supplementary Information, separately.

A list of additional formatting modifications is reported at the end of this document.

We believe that our revisions address the concerns raised by the referees and enhance the overall quality of our manuscript. We hope you will now find our work suitable for publication in "The Journal of Physical Chemistry Letters."

Yours, Sincerely,

Margherita Buraschi, Andrew Horsfield and Clotilde Cucinotta

Reviewer #1

1 The manuscript did not clearly outline the advantages of the HP DFT algorithm compared with other constant potential algorithms.

The HP-DFT algorithm exhibits its key advantage in its efficiency. The Hairy-Probes formalism was easily implemented in the DFT code by modifying only the way the occupation numbers are computed. Given that this modification constitutes the sole deviation from the regular SCF cycle, most of the required variables are already computed and provided by the rest of the DFT code. Consequently, the timing of an SCF cycle in an HP-DFT calculation does not showcase any significant slowdown with respect to the timing of an SCF cycle in standard DFT calculation. This is highlighted in the SI, in the code efficiency section.
To give a more quantitative analysis of the efficiency, the SI has been modified as follows:

- lines **8** to **16** in **Supporting_reviewed.pdf** : *“For example, a model such as the Pt(111)(6 ×6 ×3) plus water bilayer described in the paper consists of 216 Pt atoms and 24 water molecules for a total of 4080 valence electrons and 2096 occupied MO (3040 total MO, 8256 independent orbital functions). The average time per SCF cycle step for such a system was 12 circa 4.4 s for standard DFT, while the HP-DFT calculation at $\Delta\mu = 0$ eV took around 5.7 s per cycle. For both calculations, the geometry was fully relaxed in 15 optimization steps with a similar number of SCF cycle steps. Even at $\Delta\mu = 1$ eV, the average time per SCF cycle step*

was around 5.7 s. In this case, the geometry took 18 steps to fully relax. This is summarized in Figure (S1).".

- Additionally, **Figure S1**, which compares number of SCF cycles and average time per SCF cycle steps in standard DFT and HP-DFT calculations, has been added to **Supporting_reviewed.pdf**.

Another feature which makes HP-DFT a one-of-a-kind formalism is that it allows for a multi-terminal setup. Although this is not showcased in the manuscript, potentially any number of probe sets with different electrochemical potentials can be used. This paper served mostly to benchmark the formalism, so we worked on simple systems.
Furthermore, the way the HP-DFT has been implemented, the cell is charge neutral and the number of particles does not vary, ensuring that the energy is well defined.
Finally, the probes directly control the *electron* electrochemical potentials, thereby providing a proper description of the electrons in the system.

To make this point clearer, we have modified the manuscript as follows:

- lines **67** and **68** of **main_reviewed.pdf**: *"HP-DFT proved to be lightweight and highly efficient, showcasing computational costs comparable to that of standard DFT calculations.".*
- lines **162** to **170** of **main_reviewed.pdf**: *"Because of the way the HP-DFT was implemented, the cell is charge neutral. This ensures that the energy is well defined. Finally, the probes impose well-defined electron electrochemical potentials, thereby providing a proper description of the electrons in the system. Finally, another feature which makes HP-DFT a one-of-a-kind formalism is its capability to accommodate a multi-terminal setup. While exploring this aspect is beyond the scope of this manuscript, any number of probe sets with different electrochemical potentials could potentially be employed. As the focus of this paper primarily centred on benchmarking the formalism, our efforts were directed towards studying systems with a two-terminal setup for simplicity"*.
- lines **173** to **180** of **main_reviewed.pdf:** and *"Overall, the key feature of HP-DFT is its efficiency. The integration of the Hairy-Probes formalism into the DFT code was easily achieved, requiring only a modification in the way the occupation numbers are calculated. As this is the sole deviation from the standard SCF cycle, the majority of the necessary variables are readily computed and supplied by the existing DFT code infrastructure. Consequently, the use of the HP-DFT formalism in calculations did not exhibit a significant deceleration of the SCF cycle in comparison to conventional DFT algorithms. Further information regarding the code's efficiency is available in Figure S1 of the Supporting Material."* .

2 (a) In the "HP-DFT formalism" section, the authors mention a coupling strength parameter Γ_p, but this parameter is not observed in subsequent formulas. (b) It has also not been clarified how to calculate the average electron electrochemical potential $\bar{\mu}$. (c) Additionally, the authors applied μ_L and μ_R based on the assumption of $\Delta\mu_L = -\Delta\mu_R$. However, this assumption may not be appropriate, as the simulation model is not symmetrical and water molecules are present only on one side of the electrode.

(a) The HP-DFT formalism is implemented in the limit of weakly coupled probes. In the original formulation of strongly coupled probes, Γ_p appears both in the formula for the occupation numbers and in the Hamiltonian of the system. In the weak coupling limit, it is assumed that $\Gamma_p \to 0$, so this parameter does not appear in our formulas.
To clarify this point, the manuscript has been modified as follows:

lines **81** to **85** of **main_reviewed.pdf**: *"In the limiting case of probes weakly coupled to the system, $\Gamma_p \rightarrow 0$, thus it does not appear in the central formulas. It was shown that this weak coupling limit is suitable for describing EC systems as the charge is carried from one electrode to the other by ions in the electrolyte, whose diffusion rate sets the electron conduction rate between electrodes".*

(b) The average Fermi level, $\bar{\mu}$, is calculated as the average among the local Fermi levels induced by the probes' EC potential. $\bar{\mu}$ is the level which ensures charge neutrality in the system. Essentially, we set the value of the EC potential difference, $\Delta\mu = |\Delta\mu_L - \Delta\mu_R|$, and define $\bar{\mu}$ as the midpoint between the two Fermi levels. This value is then adjusted to enforce charge neutrality effectively.
To clarify this point, the manuscript has been modified as follows:
lines **113** to **115** of **main_reviewed.pdf:** *"The level $\bar{\mu}$ is calculated as the average among the local Fermi levels induced by the probes' EC potential; as such, this is the level which ensures charge neutrality in the system".*

(c) It is correct that the simulation model is not symmetrical and, therefore, the electrodes potentials would not, on their own, satisfy this relation. However, $\Delta\mu_L$ and $\Delta\mu_R$ are defined as the probes' electrochemical potentials referred to $\bar{\mu}$ and they are set by the user. In this case, therefore, it is not assumed that $\Delta\mu_L = -\Delta\mu_R$; these are the values that have been chosen to assign them.
In principle we have three electrochemical potentials values to set. We currently only use two criteria to set them (charge neutrality and applied bias), so we need an additional rule (as described above) to uniquely define all three values. We note that $\bar{\mu}$ is also used to set the EC potential for the solution probes.

To clarify this point, the manuscript has been modified as follows:
line **159** of **main_reviewed.pdf**: *"were chosen to satisfy"*

3 The interface model used in this manuscript contains a small number of water molecules only at one side electrode, while different electrochemical potentials are applied to both electrodes. In this model, water molecules may be unable to screen the excess charges on both electrodes, which could lead to serious issues with periodic boundary conditions and result in unreliable simulation results. To address this issue, the author should consider an implicit solvent model or dipole correction, or widen the electrode spacing, incorporating a sufficient number of water molecules, and conducting AIMD calculations.

While it is entirely true that there is not enough water to screen the dipole introduced by the charged slabs, this does not represent an issue for our poisson solver as periodic boundaries are not used in the direction perpendicular to the slabs' surfaces and parallel to the dipole. Instead, an implicit Posson solver is employed.
We acknowledge that this may not come across clearly in the text and we have modified the manuscript as follows:
lines **151** to **154** of **main_reviewed.pdf**: *"The introduction of an EC potential difference induces a dipole within the system. For this reason, periodic boundary conditions were applied only in the directions parallel to the surfaces of the plates (x and y), while in the direction perpendicular to them (z) an implicit Poisson solver was used.*

4 The distribution of excess charges appears to be inaccurate. In Figure 2, the atoms in the middle layers (i .e., the 2 nd and 5 th layers) carry positive charges on both positive and negative electrodes , and the amount of those charges is higher than that of the inner layers (i .e., the 3 rd

and 4 th layers). This contradicts some existing theories and computational results (Nat Commun 13, 174 , 2022 ; Nat Commun 9, 716 , 2018 ; Phys. Rev. B 7, 3541 , 1973), where the excess charges in the electrodes are primarily distributed in the inner layer, with fewer excess charges in the remaining layers.

Due to the high electronegativity of Pt, Pt(111) surfaces are negatively charged[1]. Consequently, the subsurface is positively charged. The charge then should go to zero in the middle of the slab, where bulk conditions are achieved. In a 3-layers system, such as the one discussed in Figure 2, the middle layer corresponds to the subsurface to both surfaces. Our 5-layers model is more in line with this picture (Figure 1b, top). It has to be noted that the use of Bader charges introduces some inaccuracies in the assignment of the charge to atoms. Indeed, its magnitude, is the result of the integration over atomic volumes which might include tails of the charge actually belonging to neighbour atoms, . Plotting the original average total charge density distribution along Z, however, we can see that it goes to zero in the middle of the metal slab (Figure 1, bottom), even for the three layer system.
When $\Delta\mu = 0\ eV$ the total excess Bader charge on both slabs (the sum of the charge on all layers) goes to 0.0 ± 1^{-5} |e|. When $\Delta\mu \neq 0\ eV$ the **total** excess Bader charge becomes negative on one slab and positive one the other slab. The plot of the excess Bader charge per layer reported in Figure 2 served to show that such variation happens only on the two internal surfaces, as expected for a capacitor. Since the initial charge of the internal surfaces at $\Delta\mu = 0\ eV$ is negative, these layers will only become "more negative" and "more positive" with respect to their initial state. It is the total excess Bader charge on the slabs that was used for the calculation of the capacitance.

Consequently, the HP-DFT can correctly capture the behaviour of the plate.

To clarify this, we have modified the manuscript as follows:
lines **200** to **214** of **main_reviewed.pdf**: "*When $\Delta\mu = 0\ eV$ the total excess Bader charge on both slabs (obtained from the sum of the excess Bader charge on each atom) goes to zero (within 0.0 ± 1^{-5} |e|). When $\Delta\mu \neq 0$ eV, the total excess Bader charge becomes negative on one slab and positive on the other. As can be observed in the figure, such variation in charge only occurs on the internal surfaces of the capacitor (3rd and 4th layers) as expected. Due to the high electronegativity of Pt, the atoms on the surfaces are negatively charged even at $\Delta\mu = 0$ eV. Therefore, when $\Delta\mu \neq 0$ eV the average charge per atom on the internal surfaces will become "more negative" or "more positive" with respect to the zero bias case. To compensate, the charge of the subsurface (2nd and 5th layers in our 3-layer slabs), will have a positive charge. Figure (S2e) in the Supporting Material shows the average Bader charge per atom in each layer of the plates for the Pt(111)(2 ×2 ×5) system. It can be observed that in the 5-layer plates, while the subsurfaces (2nd and 4th layers for the left plate, and 7th and 9th layers for the right plate) are still positively charged, the charge tends zero in the middle of the slab (3rd and 6th layers). This observation indicates a realistic description of the metallic slabs. The overall charge on the slabs for the Pt(111)(6 ×6 ×3) system is given in Table 2.*".

We have also added the following text and Figure to the SI:
lines **26** to **41** and **Figure S2** of **Supporting_reviewed.pdf**: "*Figures (S2a), (S2b) and (S2c) show the average Hartree potential along the Z-axis for the Pt(111)(2 ×2 ×3), Pt(111)(2 ×2 ×5) and Pt(111)(6*

---

[1] R. Khatib *et al.*, *Electrochimica Acta*, 2021, 391; M. T. Darby, C. S. Cucinotta, *Current Opinion in Electrochemistry*, 2022, 36; S. Surendralal, M. Todorova, J. Neugebauer, *Phys Rev Lett* 2021, 126, 166802.

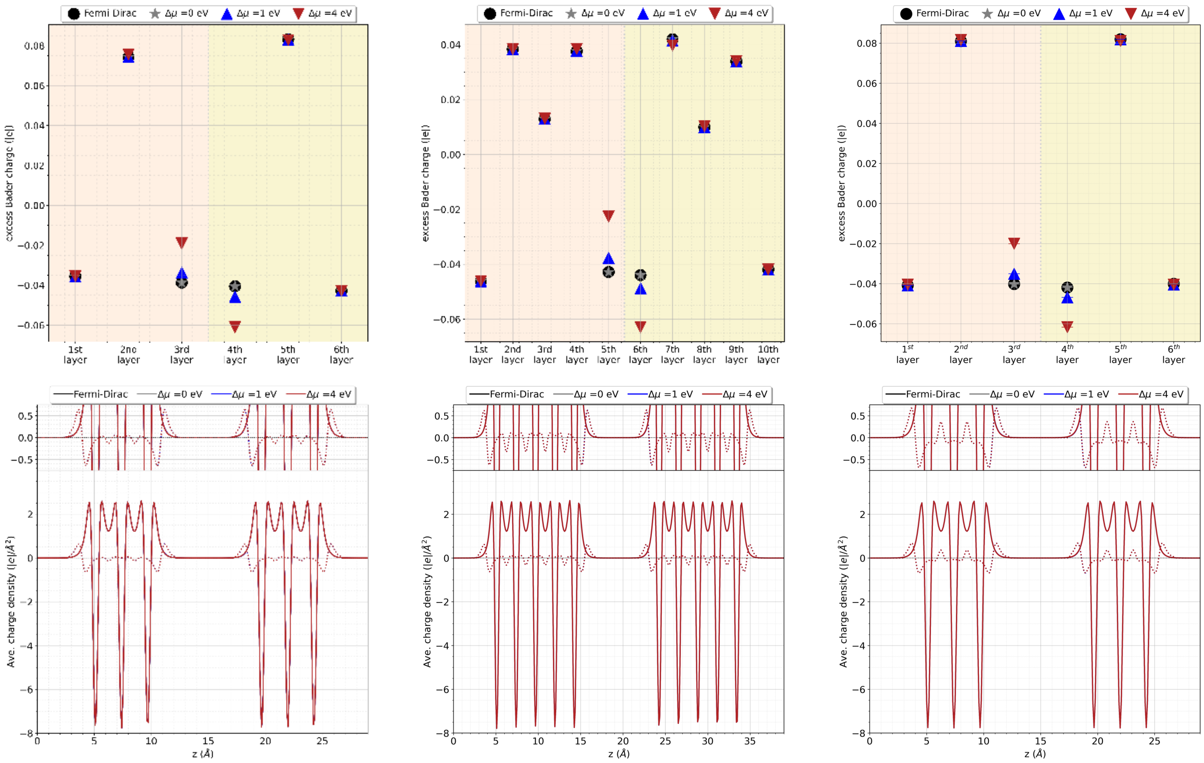

Figure 1. Average excess Bader charge per atom in each layer (top pictures) and average total charge density along the direction perpendicular to the surfaces (bottom pictures) for ***(a)*** *Pt(111)(2x2x3) system,* ***(b)*** *Pt(111)(2x2x5) system and* ***(c)*** *Pt(111)(6x6x3) system.*

×6 ×3) systems, respectively. The local Fermi levels of the left and right plates are identifiable in each model. Figures (S2d) to (S2i) illustrate the charge distribution within the plates of each model. Due to the high electronegativity of Pt, Pt(111) surfaces are negatively charged[1-3] and, as a consequence, the subsurfaces are positively charged. The charge then should go to zero in the middle of the slab, where bulk conditions are achieved. In our 3-layer systems, Figures (S2d) and (S2f), the middle layers for both slabs (2nd and 5th layers) are also the subsurfaces to both surfaces. Consequently, they are positively charged to compensate the negative charge on the surface. In our 5-layer model, while the subsurfaces (2nd and 4th layers for the left plate, and 7th and 9th layers for the right plate) are still positively charged, the charge tends zero in the middle of the slab (3rd and 6th layers), as shown in Figure (S2e). It is worth noting that the magnitude of the average charge per atom is a result of Bader analysis, which integrates over atomic volumes. Plotting the average total charge density distribution along Z, however, we can see that the total charge goes to zero in the middle of the metal slab for all systems, as shown in Figures (S2g), (S2h) and (S2i).".

5 The electrochemical stability window of water is 1.23 V, but the electrochemical potential range calculated in the manuscript is -4 eV to 4 eV, far exceeding the electrochemical window of water molecules. At such high volt ages, water molecules should have already undergone reactions.

$\Delta\mu = 4\ eV$ is the total electrochemical potential difference between the plates. Overall, the electrochemical potential applied to the left electrode (on which the water is adsorbed) with respect to $\bar{\mu}$ is either $\Delta\mu_L = -2\ eV$ or $\Delta\mu_L = 2\ eV$. To differentiate the two cases, we used $\Delta\mu = -4\ eV$ to represent the first scenario, and $\Delta\mu = 4\ eV$ to represent the second one.

We understand that this may have not come across clearly, so we have modified the manuscript as follows:
lines **235** to **239** of **main_reviewed.pdf**: *"We studied water coverage and charge redistribution at $\Delta\mu = -4\ eV, -1\ eV, 0\ eV, +1\ eV$ and $+4\ eV$. The EC potential difference is still applied such as $\Delta\mu_L = -\Delta\mu_R$. In this, negative values of $\Delta\mu$ represent negative values $\Delta\mu_L$ (i.e. for $\Delta\mu = -4\ eV$, $\Delta\mu_L = -2\ eV$ $\Delta\mu_R = +2\ eV$).".*

For water molecules to react and split there is a barrier to be overcome. Since this is a geometry relaxation at 0K, there is not enough energy to break bonds in molecules. The voltage is enough to cause the molecules to overcome the barrier to rotate to H-down and to even desorb but not to break bonds.

Reviewer #2:

6 The study conducted by Buraschi and colleagues explores the polarization effects of the electric double layer at the Pt(111)/water interface. The main novelty of this work is integrating a constant potential method originally developed by Zauchner and co-workers ((PRB, 2018, 97, 045116)) into CP2K. That method facilitates the examination of electrochemical interfaces at predetermined chemical potentials, a feature of significant interest in fields such as electrocatalysis, corrosion, and electroplating. **(a)** Despite the attractiveness of the constant potential method for modelling electrochemical interfaces, the manuscript does not clearly delineate the main advantages of the used method over existing constant-potential methods, such as those proposed by Bonnet, PRL, 2012, 109, 266101 and Bouzid, JPCL, 2018, 9, 1880. **(b)** Furthermore, the investigation into water orientation and adsorption energy in response to electrode potential, utilizing a simplified water bilayer model, does not convincingly reveal novel electrochemical insights. Consequently, this work appears to be more methodologically focused, suggesting its suitability for a journal with a technical emphasis.

(a) See our response to Q1

(b) The water bilayer system did indeed start as a simple model for proof of concept for the methodology. Its study with HP-DFT calculations, however, unveiled relevant insight for electrochemistry. Even more relevant, is that this insight came from such a simple system, without the need for computationally expensive model and/ or formalism, highlighting the importance of direct potential control.
Our study finds that the applied EC potential significantly influences coverage, interface structure and adsorption, aligning with prior research conducted through complex models and costly computational methods such as AIMD, NEGF, GC-DFT, etc. Therefore HP-DFT emerges as an efficient tool, capable of accurately capturing polarization responses with small models, thus refining our understanding of potential effects.

7 In this work, the terminology "constant potential" is used to describe the potential difference between a working electrode and a counter electrode. However, it is not the case of electrochemistry, in which the "constant potential" should be the potential difference between a working electrode and a reference electrode. I am not convinced how this method can be used for studying electrocatalysis at the so-called constant potential condition.

We recognise that "constant potential" could indeed be a misleading terminology. In the HP-DFT formalism we fix the electrochemical potential of the electrodes. We can fix the electrochemical

potential of two plates to create an electrochemical potential difference, which is a constant potential difference. This directly models the behaviour of electrochemical capacitors and junctions. In electrocatalysis the electrode potential is measured experimentally with respect to a reference electrode. To define the electrode potential drop in a half-cell - the Galvani potential, which although not experimentally measurable, can be evaluated with a calculation - it is necessary to define a reference level. Following foundational work of Trasatti and Sprik/Chang, this could be represented by the vacuum level in front of the electrolyte solution or the bulk electrolyte potential, respectively. In our system, the water layer is not sufficiently thick to effectively shield the surface dipole or provide a reference bulk electrolyte level, leading to inaccuracies in establishing such a reference level. However, this does not represent a fundamental limitation of our methodology, preventing to use it for studying the electrocatalysis of half cells at constant potential (provided that an appropriate reference point is accurately identified within the system). Despite this limitation, it is remarkable that we still observe behaviours induced by the modification of the local interfacial electric field with bias, that are consistent with what observed with much larger models. More in general, to obtain a correct description of the potential drop, two levels must be identified anyway (not only in our methodology). Our approach allows to flexibly control one of the two, adding precious flexibility to the modelling of interfacial electrochemical behaviours with respect to other methodologies. Interestingly, this reference point can be used to place the electrode potential on an absolute scale. More specifically, to accurately define a reference level, we plan to use this approach using systems with a full water layer. Further studies are ongoing. However, unravelling the intricacies of the definition of an absolute scale for the electrode potential is out of the scope of present proof-of-principle paper.

Following this comment, we have modified the manuscript, changing the terminology "constant potential" to "constant **electrochemical** potential" throughout. See also modifications introduced below.

8 The calculation of water adsorption energy, as outlined in Equation (6), raises questions. Typically, the reference state for water would be its gaseous form, making the inclusion of the last term in Equation (6) questionable. A revision or justification for this term would be beneficial.

The reviewer makes a good point about the equation we used. We have modified the formula for the adsorption energy as follows:

$$\Delta E_{ads}(\Delta\mu) = \frac{1}{n_{H2O}}[E_{system}(\Delta\mu) - E_{slab}(\Delta\mu) - n_{H2O} * E_{H2O}(gas)] \quad (1)$$

The term explicitly containing the EC potential of the slab has been removed. This is **Equation (8)** of **main_reviewed.pdf.**
The new values of adsorption energy resulted in the following plot (**Figure (5)** of **main_reviewed.pdf**):

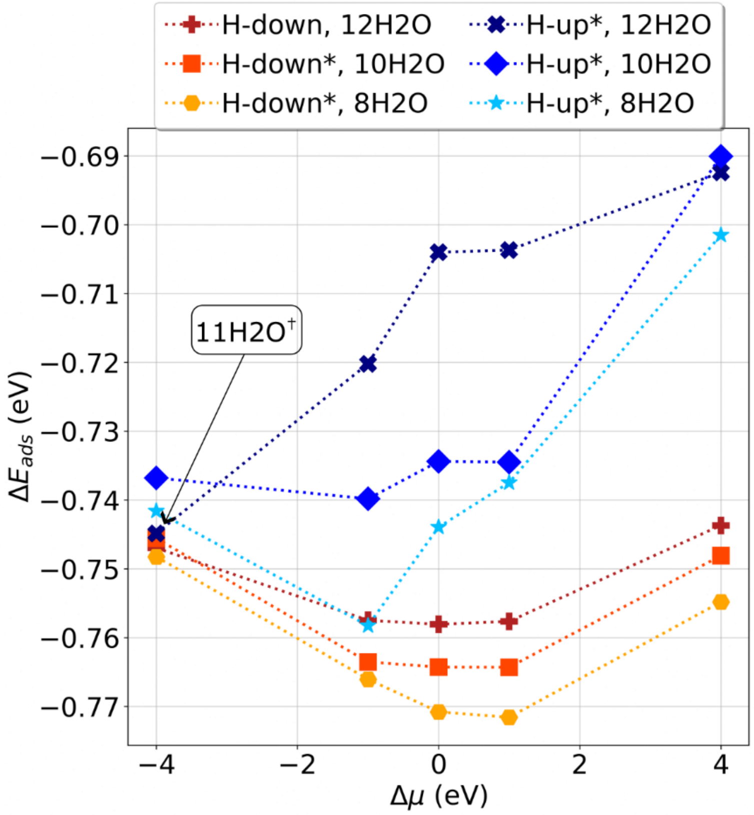

Figure 2. Adsorption energies for the water bilayer plotted as a function of the potential at different coverages: we compare both H-up and H-down configurations for cases with 12H2O, 10 H2O, and 8 H2O chemisorbed water molecules.

The new equation represents the energy for insertion of a water bilayer into the charged capacitor. The original equation stemmed from the idea that $\overline{\mu}$ could potentially be used as the electrochemical potential of the reference electrode, as discussed in Q2(c).
Consequently, the manuscript has been modified as follows:
lines **249** to **253** and lines **261** to **268** of **main_reviewed.pdf:** "*The adsorption energies for the for insertion of a water bilayer into the charged capacitor, $\Delta E_{ads}(\Delta\mu)$, were calculated according to: Equation (8), where $E_{system}(\Delta\mu)$ is the total energy of the capacitor+water system at $\Delta\mu$, $E_{slab}(\Delta\mu)$ is the energy of the slabs without water at $\Delta\mu$, $E_{H2O}(gas)$ is the energy of the molecule in gas phase and n_{H2O} is the number of adsorbed water molecules.*"

We would like to address how this methodology can be used for electrochemical applications. In EC studies the electrode potential needs to be explicitly included in the evaluation of adsorption energies. Consequently, a sensible reference level needs to be established. This can be done, for example, in situations where an extended bulk region is included in the simulation, where the potential of the bulk water could serve as the reference level. Such a level, however, does not exist in our systems, as there is not enough water to screen the dipole. Consequently, we have evaluated the adsorption energy of the water layer on a half cell, using as a reference the best evaluation for the vacuum level in our system. For the sake of this discussion, we will take the Hartree potential of in the middle of the vacuum region (where the average Hartree potential profiles for all the systems cross) as the reference value to calculate the "work functions" of the left and right electrode. Indeed, if we applied a dipolar correction in the vacuum region to cancel out the field, the resulting vacuum

level would correspond to the value of the reference level indicated in figure (4). These "work functions" therefore are calculated as:

$$W_L = V_{middle} - \mu_L \tag{2}$$

and

$$W_R = V_{middle} - \mu_R \tag{3}$$

respectively, where V_{middle} in the Hartree potential of in the middle of the vacuum region and μ_L and μ_R are the local Fermi levels on the left and right plate respectively. The inclusion of these terms in the equation for the adsorption energies then becomes:

$$\Delta E_{ads} = \frac{1}{n_{H2O}}\{E_{system}(\Delta\mu) - E_{slab}(\Delta\mu) - n_{H2O} * E_{H2O}(gas) + [q_L(\Delta\mu) * W_L(\Delta\mu) + q_R(\Delta\mu) * W_R(\Delta\mu)]\} \tag{4}$$

where $W_L(\Delta\mu)$ and $W_R(\Delta\mu)$ are the "work functions" of the left and right electrode at $\Delta\mu$ and $q_L(\Delta\mu)$ and $q_R(\Delta\mu)$ are the excess Bader charges on the left and right plate at $\Delta\mu$. The resulting adsorption energies are reported in the following plot:

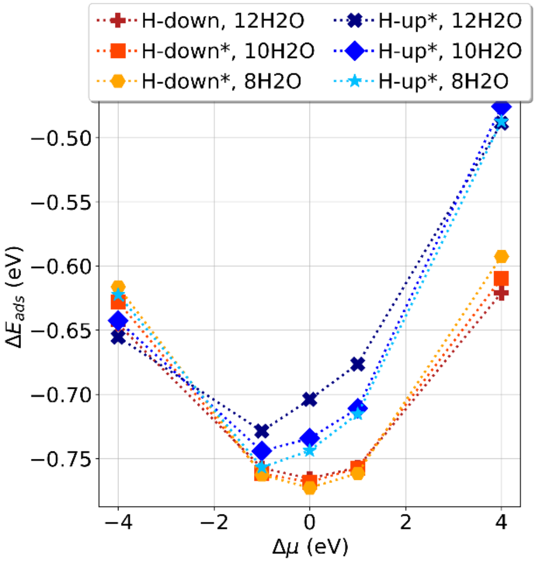

Figure 4. Adsorption energies for the water bilayer calculated with the equation (4).

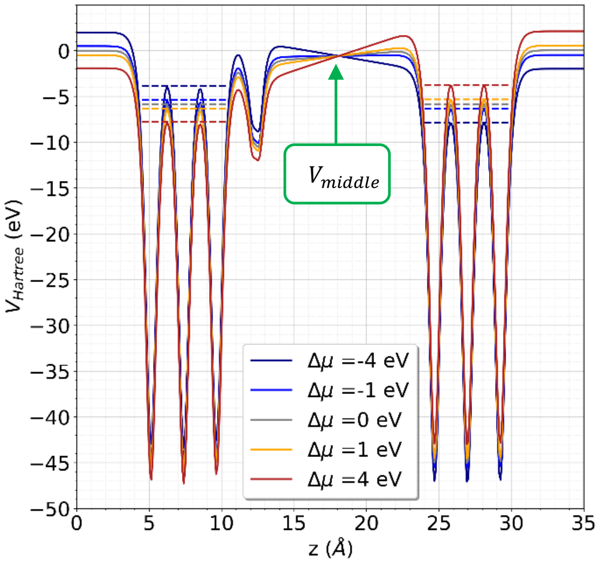

Figure 4. Hartree potentials (solid lines) and localized Fermi levels (dashed lines) for the Pt(111)(6x6x3) plus water bilayer system.

The effects of including the term between square brackets in Equation (4) are presented in Figure (3). The data in Figure (3) indicates that the low coverage H-down structures are still the most stable ones for values of $\Delta\mu$ between $-1\ eV$ and $+1\ eV$. At $\Delta\mu = 4\ eV$, however, the full coverage H-down water bilayer becomes the most stable structure. At $\Delta\mu = -4\ eV$, instead, the full coverage H-up bilayer structure is the most stable one. In this system, most of the molecules in the 2nd adsorption layer flipped from an H-up to an H-down configuration, stabilizing the bilayer. This is also the structure where one of the chemisorbed molecules in the 1st layer spontaneously desorbs from

the surface during geometry optimization. Such reduction in water coverage further stabilizes the bilayer making it more stable than its full coverage H-down counterpart. This observation aligns with recent literature in showing that capacitive response of the interface to a positive EC potential is primarily driven by the increase in surface coverage of positively charged water molecules.

In a system with a well-defined and sensible reference level, the same equations (2) to (4) can be applied using such level instead of V_{middle}.

The above discussion has been mentioned to the manuscript:
lines **284** to **296** of **main_reviewed.pdf:** *"To demonstrate the applicability our methodology to electrochemical studies, we have also evaluated the adsorption energy of the water bilayer on a half cell, using as a reference the best evaluation for the vacuum level in our system. In this case, the agreement with AIMD studies is even more remarkable, as evidenced by the increased stabilization of a higher number of water molecules at elevated potentials. This observation, in fact, aligns with recent literature in showing that capacitive response of the interface to a positive EC potential is primarily driven by the increase in surface coverage of positively charged water molecules[3,17,50]. This discussion is presented in more details in the Supporting Material."*
The detailed discussion presented above is also given in the SI, lines **114** to **148** plus **FigureS6** of **Supporting_reviewed.pdf**.

9 To better elucidate water polarization at charged surfaces, considering a water monomer model might offer a more straightforward analysis.

The analysis of the effects of the applied EC potential on a single molecule adsorbed is showcased in the Supporting Info.
We have modified the SI as follows:

- lines **50** to **54** of **Supporting_reviewed.pdf:** *"The more positive the right surface becomes the more electronic charge H2O donates to the electronegative Pt surface. Conversely, as the left electrode surface becomes more negatively charged, less electronic charge is donated to Pt; consequently, the molecule becomes less and less positively charged, going towards zero (the charge of the isolated molecule)."*.
- lines **71** to **77** of **Supporting_reviewed.pdf:** *"Figure (S4) shows the electron density difference between the system at $\Delta\mu = 4\ eV$ and the system at $\Delta\mu = 0\ eV$. An accumulation of electronic charge (indicated by the blue isosurface) between the water and the left plate, suggesting a stronger bond between the molecule and the surface. Conversely, a depletion of electronic charge (indicated by the orange isosurface) between the water and the right plate indicates a weakening of the molecule-surface bond. Both observations are in agreement with the data obtained from the Bader and geometry analysis of the system."*
- We have also added the following picture, which shows how the charge polarises under applied EC potential difference, as **Figure S4** in the Supporting Info:

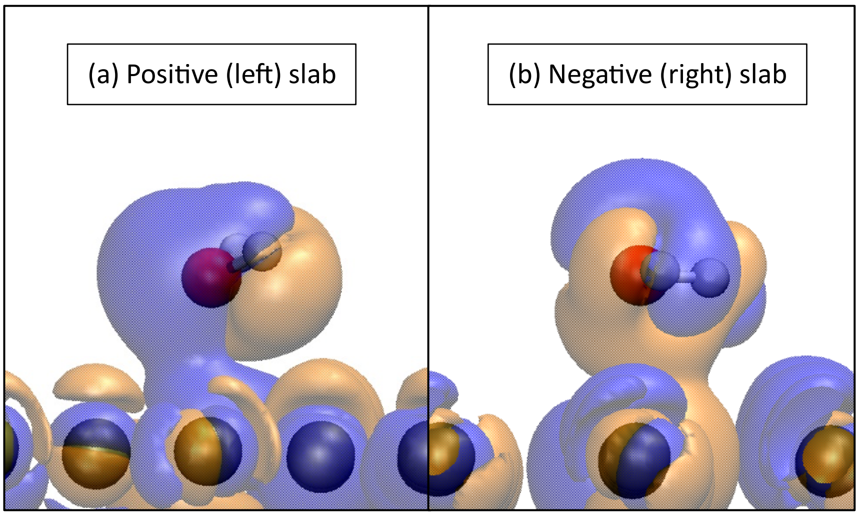

Figure 5. Electron density difference represented as isosurfaces between the system between the system at 4eV and the system at 0eV: (a) molecule on the left plate, (b) molecule on the right plate. The blue and orange surfaces represent isolevels at -0.002 e/Å² and 0.002 e/Å² respectively (negative values represent electron accumulation)

10 In presenting calculated capacitance values in Table 1, adopting the unit "uF/cm2" would facilitate easier comparison with both experimental and theoretical studies in the field.

We have modified the units in table 1 to μC and μF, **Table 2** of **main_reviewed.pdf.** We have also provided the values of capacitance per square centimetre as the reviewer suggested:
lines **221** to **223** of **main_reviewed.pdf**: *"Overall the capacitance per surface area calculated from the slab charge is 1.23 μ F/cm² and that calculated with the parallel plate capacitor formula is 1.22 μ F/cm².".*

11 The discrepancy in slab separation distances—10 angstroms for some models and 20 angstroms for others—within the method section warrants.

We used a separation between the plates of 20Å for all the systems with the water bilayer, to avoid interaction with the right surface (especially the ones in the 2nd adsorption layer).
We have modified the manuscript to explain our choice:
lines **136** to **139** of **main_reviewed.pdf**: *"to avoid interaction with the right surface (these distances are measured from the centre of mass of the atoms of the internal surfaces; to calculate the capacitance, d was corrected by the Van der Waals radius of Pt).".*

Reviewer #3

12 The authors write that the leads are typically coupled only to atoms in the contact region. Obviously, this applies to the electrode and is needed to achieve a potential drop at the interface. They also mention the implementation of "solutions probes" needed to avoid a "nonphysical situation where molecules in solution have no electrons". Do I understand correctly that this "solution leads" couple to water molecules, so that this coupling leads to a surplus/deficiency of electrons on the water molecules? What are the charges on the water molecules?

We recognise that the text may have been unclear on this point. The *main* probes needed to induce the EC potential difference are coupled to the contact regions, i.e. the electrodes. Meanwhile, the *solution* probes are indeed coupled to the atoms in the water molecules. Similarly to what done for bound states in molecular electronics, these probes are added to circumvent the problem of possible states in the system with no coupling to the leads, therefore their action would not artificially change the charge of these molecules.

The Fermi distribution for the solution probes is calculated using the average Fermi level $\bar{\mu}$ of the system (Equation (5) in the main paper). The average Fermi level is calculated as the average between the local Fermi levels induced by the probes' EC potentials. The way this level is obtained ensures charge neutrality in the system in the presence of an EC potential difference (see also response to Reviewer #1 and modifications of the paper reported therein)

We have modified the manuscript as follows:

line **94** to **97** of **main_reviewed.pdf:** *"Thus, the main probes coupled to the contact regions are used to induce an EC potential difference across the system while the solution probes coupled to the electrolyte's molecules are needed to ensure the correct population distribution in the solution."*.

13 **(a)** Some of the applied potentials are rather high and I would expect reactions to occur. Yet the shown effects are molecule desorption or reorientation. It is not clear to me, whether reactions, for example water dissociation, can occur. **(b)** How will these be affected by the "solution leads"?

(a) See our response to Q5

(b) The coupling of the solution probes is much weaker than for the main probes, so that the solution probes only influence the occupation of the electron states when the main probes are unable to. Thus, the solution leads should have little or no effect on these processes. We note that they are coupled with each atom individually and their Fermi distribution is determined using $\bar{\mu}$ (as also discussed in Q12).

14 If I understand the set-up correctly, the investigated interface consists of a Pt(111) slab in contact with two water layers, which in turn are connected to a vacuum region. **(a)** What about a bulk water region? The properties of an adsorbed water bi-layer in vacuum are not necessarily the same as the properties of an adsorbed water bi-layer in contact with water. Yet the shown results are often compared to calculations in which an extended water region is present. **(b)** Is such a comparison applicable? **(c)** Are the comparisons of timing of calculations using an extended bulk water region fare? Obviously any calculations which does not have to take care of equilibrating a bulk water region will be faster.

(a) This simplified model was chosen to establish a proof of concept, evaluating the ability of HP-DFT to model metal/water interfaces appropriately (even qualitatively). There is ongoing extension of current work for a system with an extended bulk region, however this is out of the scope of the present paper. We fully agree with Reviewer 3 observation that the properties of a water bilayer adsorbed on a surface in vacuum differ from those observed in systems covered with a full water layer. However, it is remarkable to note that with our methodology we are able to observe behaviours and trends induced by the local interfacial electric field that are consistent with what observed with much larger models. See also reply to Reviewer 2 for a discussion of this point.

(b) Of course, the reviewer is correct. The behaviour of water at the interface is indeed influenced by the presence or absence of a bulk region. The primary aim here was to show that, albeit starting as a simple proof of concept, our simplified model manages to *qualitatively* capture the behaviour expected at a metal/water interface under bias and that local effects from charge polarization can be recovered without resorting to very large model systems. We acknowledge that further studies involving an extended bulk water region are necessary for a more comprehensive understanding of the Pt-water interface under bias, but this is not the scope of our paper.
To make this clear we have modified the manuscript as follows:
lines **352** to **356** of **main_reviewed.pdf:** *"Of course, one can expect that the presence of an extended bulk layer would affect the behaviour of water at the interface. Nonetheless, despite starting as a simple proof of concept, our simplified model manages to qualitatively capture the expected behaviour at a metal/water interface under bias, successfully accounting for local effects stemming from charge polarization."***.**

(c) The timing reported in the SI refers to the system with a single water molecule. We have modified this and given the information for the water bilayer system in the "**Code efficiency**" section of the Supporting Info. See our reply to Q1 for more detail.

15 I find the discussion of "The response of a water bilayer to the charge of electrode potential" to be a bit selective on the comparison to available literature and selection of which articles to cite. The understanding of electrochemical interfaces has evolved since the cited 2004 article and people are aware that changes in the electrode potential affect the polarisation, orientation and adsorption/desorption behaviour of water molecules at the interface, even if electron transfer reactions have not yet occurred.

We acknowledge the evolution in the understanding of EC interfaces since the publication of the 2004 paper. This understanding has been achieved using large and complex models, along with sophisticated methodologies, resulting in costly calculations. What we propose is an alternative methodology capable of yielding comparable outcomes at much reduced computational effort. This paper serves as a benchmark for a methodology which is highly efficient and therefore extremely promising for applications in AIMD studies.
That said, in response to the observation of Reviewer 3, we have expanded our list of citations in the hope to provide a broader spectrum of relevant sources:
line **297** of **main_reviewed.pdf:**
Gross *et al.* 2022 (https://doi.org/10.1021/acs.chemrev.1c00679), **Gross *et al.* 2020** (https://doi.org/10.1039/C9CP06584A), **Le *et al.* 21** (https://doi.org/10.1021/jacsau.1c00108) and **Otani *et al.* 2008** (https://doi.org/10.1039/B803541E)**.**

16 In the discussion of the set-up the authors mentioned two numbers for the separation between the Pt slabs – 10 Ang and 20 Ang. It is not clear to me, whether the separation of 20 Ang applies to all the models containing water or only to the ones with a reduced number of water molecules. It is also not clear why it was necessary to change the separation length.

The separation length between the plates has been increased to 20 Å for all the systems containing the water bilayer (either full or undercoordinated). This was done simply to ensure that the water molecule in the second adsorption layer would not interact with the other surface.
See our response to Reviewer 2, Q11, for a more detailed discussion, also reporting the modifications to the manuscript in response to this point.

17 Why is an entropy term included only for the gas-phase molecule? What about the water molecules at the interface? What about entropic contribution by the bi-layer water molecules? Surely this will be lower, but not necessarily negligible. Do the authors have an estimate about their magnitude and whether neglecting them is justified?

The reviewer makes a good point. After double checking the literature, however, we found that the energy of adsorption of a water bilayer on a metal surface is generally reported to be as adsorption energy ΔE_{ads} rather than free energy of adsorption ΔG_{ads} [2]. To make our data comparable with the literature, we have elected to use this quantity in the manuscript as well. Consequently, we have modified the manuscript. See our response to Q8 also reporting about the modifications to the paper.
We would like to highlight that in this proof-of-concept paper we are interested in describing trends of adsorption energy as a function of potential and coverage and we expect that the entropic contribution would not change them.

Indeed, we have evaluated ΔG_{ads} with an estimation of the entropic contribution for the adsorbed water bilayer as well:

$$\Delta G_{ads}(\Delta\mu) = \frac{1}{n_{H2O}}[G_{system}(\Delta\mu) - E_{slab}(\Delta\mu) - n_{H2O} * G_{H2O}(gas)]$$

where $G_{system}(\Delta\mu) = E_{system}(\Delta\mu) - TS$ and $G_{H2O}(gas) = E_{H2O}(gas) - TS$. The entropic contribution for the water molecule in gas phase was taken from Thrush *et al.* 2016 (https://doi.org/10.1021/acs.jpcb.6b01690). The value of the entropic correction for the water bilayer adsorbed on the surface, instead, was taken as an estimation of the upper bound from Braunwarth *et al.* 2022 (https://doi.org/10.1002/cphc.202200336). The values of the free energy are reported below:

[2] A. Michaelides *et al., Physical Review B*, 2004, 69; S. Schnur and A. Gross, *New Journal of Physics*, 2009, 11; K. Tonigold and A. Gross, *Journal of Computational Chemistry*, 2012, 33.

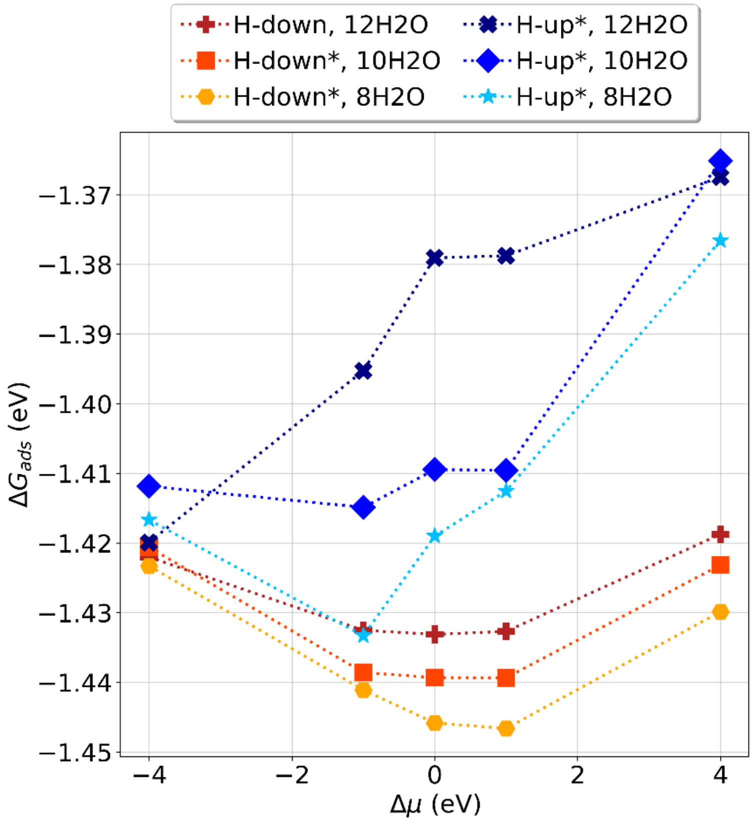

Figure 6. Adsorption energies for the water bilayer plotted as a function of the potential at different coverages: we compare both H-up and H-down configurations for cases with 12H2O, 10 H2O, and 8 H2O chemisorbed water molecules.

As can be observed from Figure (6), the overall trend remains the same as that of the ΔE_{ads} (Figure(2)). Therefore, our discussion on the structure and stability of the bilayer remains unaffected.

18 I have difficulties with the formulation "the potential increases" (p.11). I assume the authors mean "becomes more positive/negative"?

Yes, reviewer 3 is correct. We have modified the manuscript to make this point clearer. See our reply to Q5 for the modifications to the manuscript.

19 What does a "full coverage water bilayer" mean – a 12 water molecules structure in the first and 12 water molecules in the second layer? What is the meaning of "low water coverage structures"? Please be more precise.

As correctly pointed out by the reviewer, the full coverage water bilayer is the system with 12 molecules on the 1st and 12 molecules in the 2nd adsorption layer. This system is defined "full coverage" as 12 is the maximum number of molecules which can be chemisorbed on our structures (6x6 surface) for a H-down and H-up bilayer structure. The "low water coverage structure" are those were some of the water molecules were removed from the 1st adsorption layer.
We have modified the manuscript to make this more understandable:
lines **131** to **135** of **main_reviewed.pdf**: *"This system is defined as "full coverage" as 12 is the maximum number of molecules which can be chemisorbed on the 6x6 surface for an ice-like H-down*

and H-up bilayer structure. Additionally, we created "low water coverage structures" by removing 2 and 4 water molecules from the 1st water layer in both H-up and H-down models. These structures are denoted as 12H2O, 10H2O and 8H2O.".

20 I believe that a citation is missing on p.12 when the authors say that their "results also align with recent literature".

We have added the necessary citations:
line **295** of **main_reviewed.pdf**
Khatib *et al* 2021 (https://doi.org/10.1016/j.electacta.2021.138875), **Darby *et al* 2022** (https://doi.org/10.1016/j.coelec.2022.101118) and **Le *et al* 2020** (https://doi.org/10.1126/sciadv.abb1219).

21 What is the meaning of E_h?

E_h refers to Hartree energy, the standard units used in many computational codes; it is the unit of energy in the atomic units system.

22 Please be more specific when writing that the "HP-DFT formalism in calculations did not exhibit a significant deceleration of the SCF cycle." Some numbers were provided in the SI comparing timings for one cycle. What is not clear to me is, how long does the overall equilibration of the system take. After all, one needs to equilibrate the various leads. How efficient the overall performance is, would likely very much depend on the implementation and used algorithm.

See our response to Q1 and Q14(c) for additional detail on code efficiency.

In addition, we would like to clarify that the leads are not a physical part of the system as in NEGF approaches, so they don't need to be equilibrated (A HP-DFT calculations is done in one step).

23 While I can guess which DOS in Fig. 2b relates to the left and which to the right lead, it would be helpful to also write this explicitly in the figure.

We have modified the figure according to the reviewer's suggestion. **Figure (3b)** of **main_reviewed.pdf.**

24 It would be helpful to incorporate the labels of the graphs in Fig. 4b into the figure.

We have modified the Figure according to the reviewer's suggestion. **Figure (6b)** of **main_reviewed.pdf.**

25 How are distances measured? Do the authors use the centre of mass of a water molecule or maybe the oxygen in a water molecule?

The distances are measured from the centre of mass of the inner layers of the plates. The distance used for the calculation of the capacitance, however, has been corrected for the Van der Walls radius. Following the reviewer's question, we have clarified this point in the. See our response to Reviewer 2, Q11, also reporting our modifications to the manuscript.

Additional Modifications

26 Supporting Information Statement: A brief, non sentence description of the actual contents of each supporting information file is required. This description should be labelled Supporting Information and should appear before the Acknowledgement and Reference sections. Examples of sufficient and insufficient descriptions are as follows:
*Examples of sufficient descriptions: “Supporting Information: 1H NMR spectra for all compounds” or “Additional experimental details, materials, and methods, including photographs of experimental setup”.
*Examples of insufficient descriptions: “Supporting Information: Figures S1-S3” or “Additional figures as mentioned in the text”.

An additional document named “Supporting_Information_Statement.tex” with the required information has been added to the zip file. Consequently, the following text has been added to the manuscript:
lines **363** to **366** of **main_reviewed.pdf:** “*Supporting Information: Additional detailed data for each of the systems discussed, including Tables and Figures. Additional analysis of a water monomer model on a charged Pt(111) surface, with corresponding discussion, Tables and Figures. Further discussion about the application of the methodology discussed for computational electrochemistry studies.*”**.**

27 Abstract: Avoid using numbered citations in the Abstract. If references are required, present them in-line, formatted in ACS style [e.g. Journal Name Abbreviation (italics) Year (bold), Volume Number (italics), Full Page Range (or Single Article Number)]. All references should start with the number 1 in the main text (not the Abstract).

We have modified the reference in the abstract as required, in line **8** of **main_reviewed.pdf.**

28 Headers: Remove the section heading(s) throughout the body of the manuscript (you can leave Methods, Abstract, and TOC Graphic headings).

The headings have been removed from the manuscript as required.

29 TOC Graphic: Please resize the TOC graphic per journal guidelines (2 in x 2 in) and move to the correct position (on the same page as the abstract).

The TOC graphic has been modified, resized, and moved according to the guidelines. **Figure (1)** of **main_reviewed.pdf.**

30 References: In both the main file and the supporting information, fix the style of all references to use JPCL formatting (check all references carefully). ***JPC Letters reference formatting requires that journal references should contain: () around numbers; author names; article title (titles entirely in title case or entirely in lower case); abbreviated journal title (italicized); year (bolded); volume (italicized); and pages (first-last). Book references should contain author names; book title (in the same pattern); publisher; city; and year. Websites must include date of access. -Journal name abbreviation needed in some refs -Titles in Title and lower case in some refs - Missing page range in some refs

The References have been modified according to the requested format.

31 Supporting Information: Please number SI pages in the following format: “S1, S2…”

The numbering of the pages in the SI has been modified according to the requested format.

32 Further Modifications:

- we have modified **Equation (4)** in line **106** of **main_reviewed.pdf.** We noticed a typo in the denominator.

jz-2023-03615x.R2

Name: Peer Review Information for "Revealing Interface Polarisation Effects on the Electrical Double Layer with Efficient Open Boundary Simulations under Potential Control"

Second Round of Reviewer Comments

Reviewer: 1

Comments to the Author

I think the authors have addressed satisfactorily the comments from the reviewers. In particular, the added data and discussions strengthen the conclusion. Then, I recommend the revised manuscript be published.

Author's Response to Peer Review Comments:

Dear Editor,

We are delighted that you are ready to accept our submission after the indicated non-scientific changes.

All the coloured text highlights have now been removed and a clean" version of the manuscript and Supporting Information files has been uploaded. We have also adjusted the figure designations to correctly sequence the TOC and figures as per your instructions.

We are looking forward to have our manuscript published in the Journal of Physical Chemistry Letters.

Best Regards,

Clotilde Cucinotta
